# A J-Protein Co-chaperone Recruits BiP to Monomerize IRE1 and Repress the Unfolded Protein Response

**DOI:** 10.1016/j.cell.2017.10.040

**Published:** 2017-12-14

**Authors:** Niko Amin-Wetzel, Reuben A. Saunders, Maarten J. Kamphuis, Claudia Rato, Steffen Preissler, Heather P. Harding, David Ron

**Affiliations:** 1 Cambridge Institute for Medical Research, University of Cambridge, Cambridge CB2 0XY, UK

**Keywords:** Allosteric Regulation, Biological Feedback, Endoplasmic Reticulum, Fluorescence Resonance Energy Transfer, HSP70 Heat-Shock Proteins, Stress, Protein Dimerization, Protein Folding, Repressor Protein, Unfolded Protein Response

## Abstract

When unfolded proteins accumulate in the endoplasmic reticulum (ER), the unfolded protein response (UPR) increases ER-protein-folding capacity to restore protein-folding homeostasis. Unfolded proteins activate UPR signaling across the ER membrane to the nucleus by promoting oligomerization of IRE1, a conserved transmembrane ER stress receptor. However, the coupling of ER stress to IRE1 oligomerization and activation has remained obscure. Here, we report that the ER luminal co-chaperone ERdj4/DNAJB9 is a selective IRE1 repressor that promotes a complex between the luminal Hsp70 BiP and the luminal stress-sensing domain of IRE1α (IRE1^LD^). *In vitro*, ERdj4 is required for complex formation between BiP and IRE1^LD^. ERdj4 associates with IRE1^LD^ and recruits BiP through the stimulation of ATP hydrolysis, forcibly disrupting IRE1 dimers. Unfolded proteins compete for BiP and restore IRE1^LD^ to its default, dimeric, and active state. These observations establish BiP and its J domain co-chaperones as key regulators of the UPR.

## Highlights

• The endoplasmic reticulum co-chaperone ERdj4 selectively represses IRE1 signaling
• ERdj4 associates with the IRE1 luminal domain and recruits the Hsp70 BiP
• Recruited BiP hydrolyzes ATP to disrupt the active IRE1 luminal domain dimer
• Unfolded proteins compete for the repressive machinery to restore IRE1 dimers

## Graphical Abstract

**Figure:**
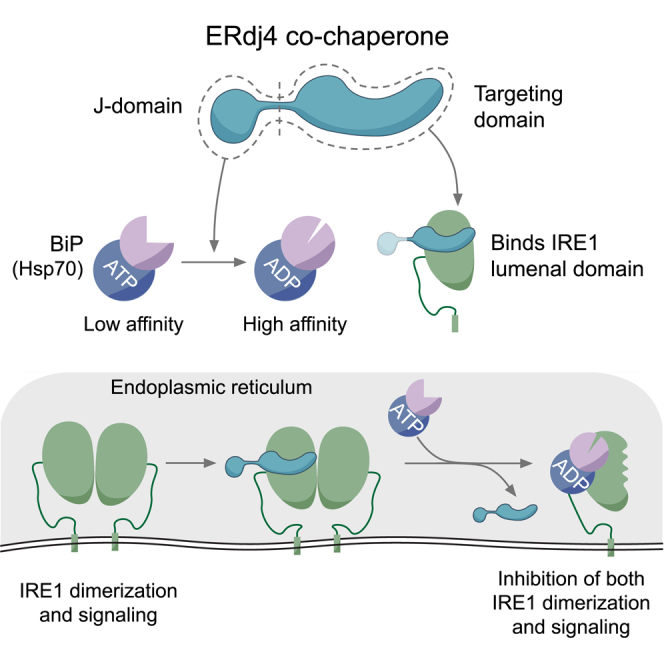

## Teaser

Molecular basis for the regulation of the unfolded protein response by chaperones and misfolded proteins.

## Introduction

Secretory and transmembrane proteins enter the endoplasmic reticulum (ER) as unfolded polypeptides and emerge as folded and processed proteins. The protein folding capacity of the ER, as measured by its luminal volume and the levels of its protein-folding and processing machinery, is matched to the inward flux of secretory and transmembrane proteins by an unfolded protein response (UPR) (Cox et al., 1997, Kozutsumi et al., 1988). A stress signal arising from an imbalance between unfolded proteins and the ER machinery is recognized by three ER-localized transmembrane proteins—IRE1, PERK, and ATF6—that affect a rectifying transcriptional and translational response to restore protein-folding homeostasis (reviewed in Walter and Ron, 2011). Although a good deal is known about the effector functions of the UPR transducers, the physiological significance of ER stress, and the response to it (reviewed in Wang and Kaufman, 2016), the upstream molecular mechanisms that detect the ER stress signal remain poorly understood.

IRE1, the most conserved and studied UPR transducer (Cox et al., 1993, Mori et al., 1993), detects stress with its ER-luminal domain (IRE1^LD^) and dimerizes, leading to dimerization-dependent autophosphorylation of its cytosolic domain (Shamu and Walter, 1996) and the subsequent activation of its cytosolic endoribonuclease activity (Lee et al., 2008). Activated IRE1 unconventionally splices the mRNA of the transcription factor *XBP1*/*HAC1* (Calfon et al., 2002, Cox and Walter, 1996, Yoshida et al., 2001), promoting *XBP1* translation and a conserved *XBP1*-dependent gene-expression program.

Dimerization emerges as a key upstream event both in IRE1 activation and in activation of PERK, which shares with IRE1 a structurally related ER-stress-sensing luminal domain (Harding et al., 1999, Carrara et al., 2015a). Two hypotheses have been put forth to explain the coupling of ER stress to dimerization. In one hypothesis, unfolded proteins are proposed to serve as activating ligands by directly binding to the luminal domain and stabilizing it in a dimeric conformation. An alternative hypothesis holds that the UPR is organized along principles similar to its cytosolic counterpart, the heat shock response, in which an imbalance between unfolded proteins and heat shock protein (Hsp)70 chaperones is recognized via the former’s ability to compete for the latter, kinetically disrupting repressive complexes between chaperones and stress transducers (Abravaya et al., 1992, Tomoyasu et al., 1998).

The IRE1 luminal domain crystallizes as a dimer, which, in the yeast protein, is traversed by a groove that could engage an extended peptide as a stabilizing ligand (Credle et al., 2005). Yeast IRE1^LD^ peptide ligands have been identified, but when added to solutions of yeast IRE1^LD^, they principally affect a transition from a collection of oligomers to higher-order oligomers (Gardner and Walter, 2011). In the crystal structure of mammalian IRE1^LD^ and the related PERK^LD^, the groove is occluded (Carrara et al., 2015a, Zhou et al., 2006). Furthermore, mammalian IRE1^LD^ is a dimer even in pure, dilute conditions (Liu et al., 2000, Zhou et al., 2006). Thus, the role of unfolded protein binding in promoting the monomer-to-dimer transition that initiates UPR signaling remains unclear.

The ER lumen has a single Hsp70 chaperone, BiP. Reversible chaperone repression as the regulatory principle of UPR activity is supported by an inverse correlation between IRE1 activity and the amount of the ER-localized BiP recovered in complex with it (Bertolotti et al., 2000, Oikawa et al., 2009, Okamura et al., 2000), a feature that extends to the related UPR transducer PERK (Bertolotti et al., 2000, Ma et al., 2002). Furthermore, mutations in yeast BiP (Kar2p) that stabilize the BiP-IRE1^LD^ interaction repress the UPR (Kimata et al., 2003), and only unfolded proteins that engage BiP can induce the UPR (Ng et al., 1992). However, beyond plausibility suggested by these correlative cell-biological findings and the argument of evolutionary analogy, this model was otherwise unsupported.

Hsp70 chaperones undergo a nucleotide-binding and hydrolysis-dependent cycle that dramatically alters their affinity for substrates. The intrinsic ATPase activity of Hsp70s is low, and ATPase-accelerating J-protein co-chaperones are therefore required for efficient substrate recognition and binding by Hsp70 (reviewed in Kampinga and Craig, 2010, Mayer, 2013). This feature arises from the ability of the co-chaperones to stimulate the ATPase activity of the Hsp70 via their conserved J domains while presenting diverse substrates to the Hsp70 via their divergent targeting domains. As a consequence, the Hsp70 initially interacts with substrates in a high-*k*_on_, ATP-bound state and then retains the substrate in a low-*k*_off_, ADP-bound state. Cycles of substrate release, accelerated by nucleotide exchange factors (NEFs, reviewed in Behnke et al., 2015) and rebinding, specified by the J domain co-chaperone, result in substrate-selective ultra-affinity (De Los Rios and Barducci, 2014, Misselwitz et al., 1998) that is the basis for formation of Hsp70-substrate complexes. Here, we drew on these well-established principles to examine the possibility that past failures to obtain biochemical support for reversible BiP-mediated repression as the basis for UPR regulation arose from the absence of a suitable ER-localized co-chaperone in the experimental systems used.

## Results

### ERdj4 Selectively Represses IRE1 Signaling in Mammalian Cells

**Figure 1:**
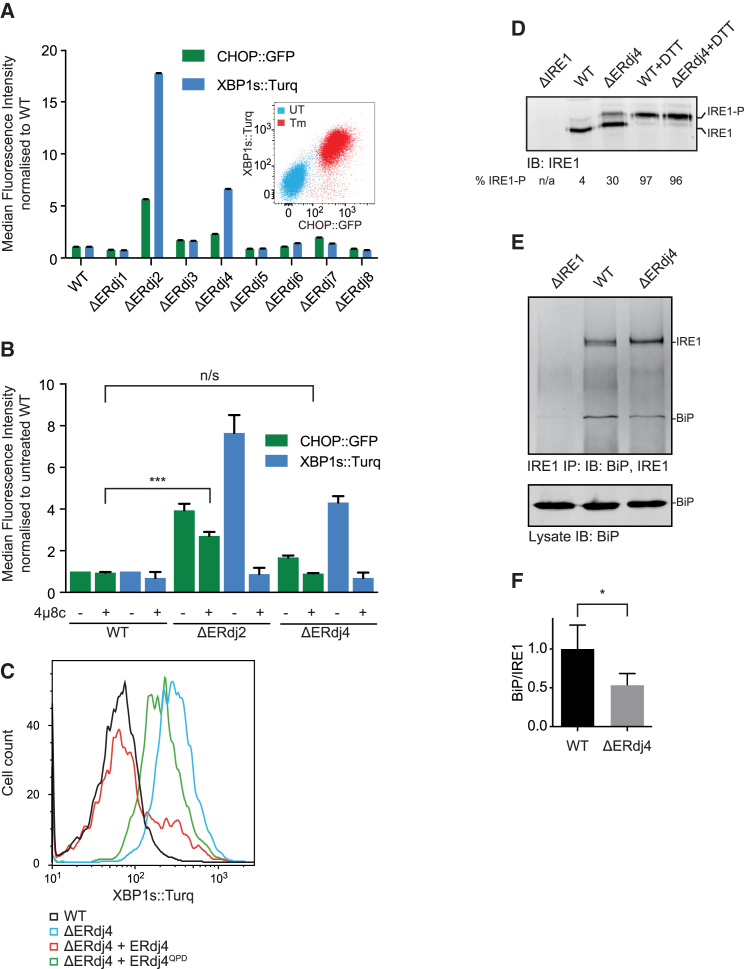
ERdj4 Is a Selective IRE1 Repressor (A) XBP1s::Turquoise and CHOP::GFP-reporter activity in CHO cells with the indicated ER-localized J-protein (ERdj) deleted. Shown is the median fluorescence (± SEM) from 20,000 cells, normalized to WT. Inset: 2-dimensional flow cytometry of untreated (UT) and tunicamycin-treated (Tm) WT CHO reporter cells. (B) XBP1s::Turquoise and CHOP::GFP activity in CHO cells untreated or treated with the IRE1 inhibitor 4μ8C, which blocks IRE1-dependent CHOP activation. Fluorescence normalized to WT. Mean of medians ± SD, n = 3, ^∗∗∗^p = 0.0005, repeated measurements one-way ANOVA, Dunnett’s multiple corrections test. (C) XBP1s::Turquoise signals from cells transfected with empty plasmid or with mCherry marked plasmid encoding ERdj4 with a WT or inactive J domain (ERdj4^QPD^). Transfected cells were gated for moderate mCherry expression levels, as shown in Figure S1B. (D) Immunoblot of immunoprecipitated endogenous IRE1α analyzed by Phos-tag SDS-PAGE. Where indicated, cells were treated with dithiothreitol (DTT). Fraction of active (phosphorylated) IRE1-P from this representative blot is noted. (E) Representative immunoblot of endogenous IRE1α and associated BiP recovered from the indicated cell lines by immunoprecipitation of IRE1α. (F) Ratio of BiP to IRE1 signal in six independent experiments as in (E). Mean ± SD, ^∗^p = 0.0118, parametric ratio paired Student’s t test). See also Figure S1.

Hsp70s are recruited to their substrates by J-domain-containing co-chaperones (J-proteins). To examine the potential role of ER-localized J-proteins (ERdjs) in recruiting BiP to IRE1 to repress signaling, we used CRISPR-Cas9 genome editing to systematically inactivate the genes encoding the eight known ERdjs in Chinese hamster ovary (CHO) cells that expressed an XBP1s::Turquoise reporter of IRE1 RNase activity and a CHOP::GFP reporter primarily under the control of PERK. The two reporters responded in parallel to drug-induced unfolded protein stress (Figures 1A, inset, and S1A). Deletion of ERdj2 (Sec63) strongly activated both reporters (Figure 1A), consistent with a role for this co-chaperone in supporting BiP-mediated ER-protein-folding homeostasis or in repression of both IRE1 and PERK. In contrast, deletion of ERdj4 preferentially activated the XBP1::Turquoise reporter. The minor activation of CHOP::GFP observed in the ΔERdj4 cells was completely suppressed by treatment with the IRE1 inhibitor 4μ8c (Figure 1B), indicating that it arose not from PERK activation but rather from IRE1’s downstream contribution to *CHOP* induction (Wang et al., 1998). Together, these observations suggest that, unlike ΔERdj2, activation of the IRE1 branch by ΔERdj4 is unlikely to reflect solely compromised ER protein folding.

**Figure S1:**
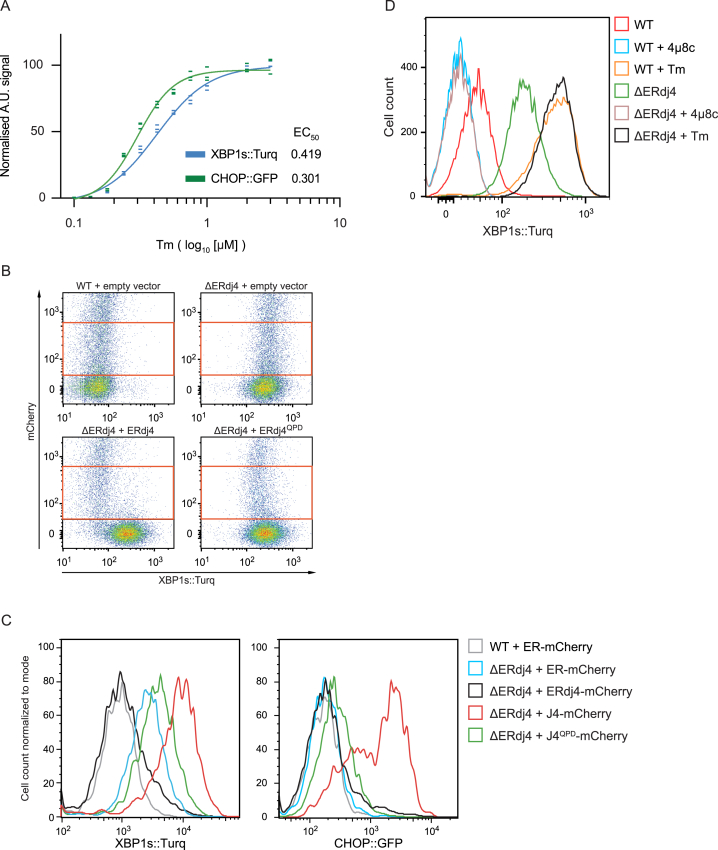
Wild-Type ERdj4 Rescues ΔERdj4, Related to Figure 1 (A) Plot of tunicamycin (Tm) concentration-dependent changes in XBP1s::Turquoise and CHOP::GFP reporter gene activity in wild-type CHO cells. Shown is the median fluorescence value (normalized to the untreated sample) obtained from 10,000 cells in experimental triplicates and the fit to a sigmoidal dose-response curve. (B) Dual channel flow cytometry plots of the XBP1s::Turquoise reporter and mCherry (a transfection marker) in wild-type and ΔERdj4 cells transiently transfected with a mCherry-tagged plasmid encoding no ERdj4 (“empty”), wild-type ERdj4 and mutant ERdj4^QPD^. The red rectangle delineates the gate used to select cells expressing moderate levels of mCherry-tagged plasmid for the histogram shown in Figure 1C. (C) XBP1s::Turquoise and CHOP::GFP signals from cells of the indicated genotype (wild-type, WT or ΔERdj4) transfected with ER-localized mCherry (ER-mCherry, a control) or mCherry tagged full-length ERdj4 (ERdj4-mCherry), mCherry tagged ERdj4 isolated J domain (1-90) (J4-mCherry; WT and QPD, lacking the C-terminal targeting domain). Transfected cells were gated for moderate mCherry expression as in (B) above. (D) XBP1s::Turquoise signals from wild-type or ΔERdj4 cells. Where indicated, cells were treated with tunicamycin (Tm) or the IRE1 inhibitor 4μ8c.

The ability of ERdj4 to repress IRE1 was dependent on the integrity of its J domain and its C-terminal targeting domain. Wild-type (WT) ERdj4 attenuated IRE1 activity in ΔERdj4 cells, but H54Q mutant ERdj4 (ERdj4^QPD^, disrupting the motif required for productive interactions with Hsp70, Wall et al., 1994) was largely inert (Figures 1C and S1B). Expression of an ER-localized truncated ERdj4 fused to mCherry (ERdj4 residues 1–90, containing the J domain, but lacking the C-terminal targeting domain), failed to attenuate IRE1 activity in ΔERdj4 cells and instead further activated both the IRE1 and PERK reporters (Figure S1C). This feature required integrity of the J domain (compare the red and green traces in Figure S1C) and is consistent with the ability of a deregulated J domain to perturb protein-folding homeostasis in the ER. Phosphorylation of endogenous IRE1α was consistently higher in ΔERdj4 CHO cells but increased further during ER stress (Figure 1D), a feature shared by the XBP1::Turquoise reporter (Figure S1D). These findings are consistent with a J-domain- and targeting-domain-dependent role for ERdj4 in the repression of endogenous IRE1 signaling.

A special relationship between ERdj4 and the IRE1 branch of the UPR is further suggested by the selective regulation of the *ERdj4* gene by IRE1/XBP1 activity (Adamson et al., 2016, Lee et al., 2003) and by the phenotype of ERdj4 inactivation in mice, which mimics XBP1 overexpression (Fritz and Weaver, 2014). The notion that ERdj4 may repress IRE1 directly was further supported by evidence that it plays a role in formation of the (hypothesized) repressive BiP-IRE1^LD^ complex: the amount of BiP recovered in complex with endogenous IRE1α from ΔERdj4 cells was reduced by half relative to the WT cells (Figures 1E and 1F).

### ERdj4 Promotes Association of BiP with the Structured Core Region of the IRE1 Luminal Domain in Cells

**Figure 2:**
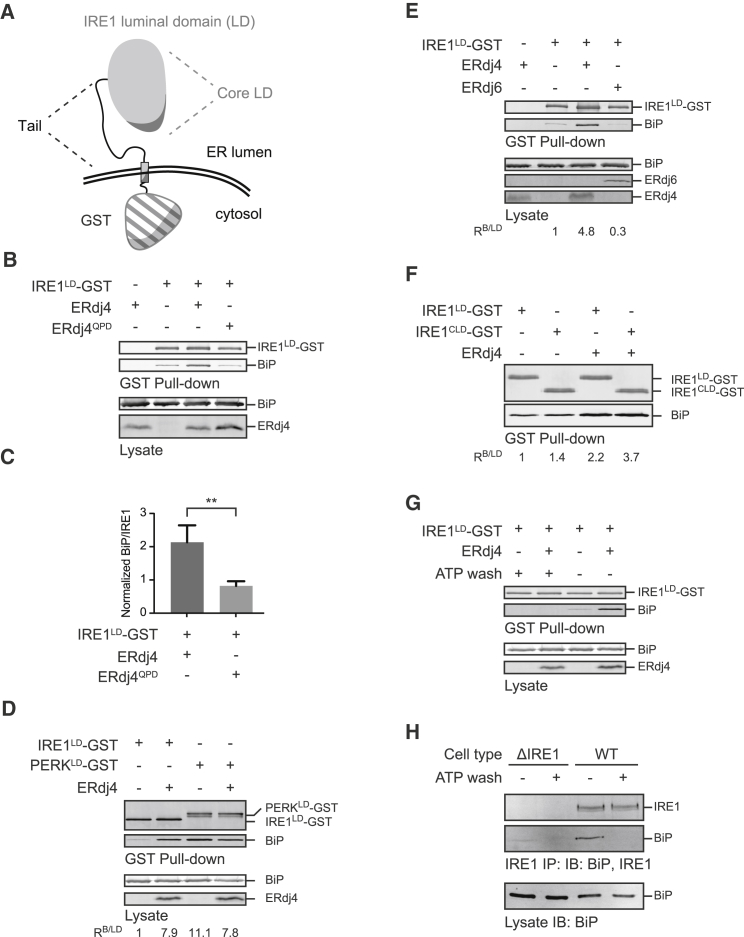
ERdj4 Promotes a BiP-IRE1 Complex (A) Schema of the IRE1^LD^-GST protein containing the entire human IRE1α luminal and transmembrane domains (residues 19–486, solid) fused to GST (striped). (B) Representative immunoblots of IRE1^LD^-GST and endogenous BiP, recovered by glutathione affinity chromatography or in lysate of transfected ΔERdj4 cells. (C) Ratio of BiP to IRE1^LD^-GST signal from 4 experiments, as in (B). Mean ± SD. ^∗∗^p = 0.0048, parametric ratio paired Student’s t test). (D) As in (B); compares IRE1^LD^-GST to PERK^LD^-GST. R(B/LD) notes the ratio of the BiP signal to the LD-GST species from the representative experiment shown. (E) As in (B); compares ERdj4 to ERdj6. (F) As in (B); compares IRE1^LD^-GST to IRE1^CLD^-GST. (G) As in (B); prior to elution with sample buffer, the indicated glutathione Sepharose beads were incubated for 5 min with 3 mM ATP at room temperature. (H) Immunoblot of endogenous IRE1α and BiP recovered from CHO cells of the indicated genotype by immunoprecipitation of IRE1α. Prior to elution with sample buffer, the indicated protein-A Sepharose beads were incubated with ATP (as in G). The bottom panel shows the input of BiP in the two samples.

To further probe ERdj4’s role in BiP recruitment to IRE1^LD^, we replaced the cytosolic effector domain of IRE1 with glutathione S-transferase (GST), yielding a convenient sensor comprised of IRE1α’s luminal and transmembrane domain fused to cytosolic GST and uncoupled from downstream signaling (Figure 2A). IRE1^LD^-GST recovered by glutathione affinity chromatography from *ΔERdj4* cells was associated with some BiP. However, in *ΔERdj4* cells, co-transfection of IRE1^LD^-GST with WT ERdj4 increased the recovery of BiP by 2.5-fold compared with co-transfection of ERdj4^QPD^ (Figures 2B and 2C). ERdj4 expression did not increase the recovery of BiP in complex with the luminal domain of PERK (Figure 2D). ERdj6, another UPR-induced ER-localized J-protein, did not increase recovery of BiP in complex with IRE1^LD^-GST (Figure 2E). ERdj4 thus has a specific capacity to promote a BiP-IRE1^LD^ complex.

The luminal domain of IRE1 consists of a structured core region (CLD, core luminal domain) and a presumably unstructured tail region leading to the transmembrane domain (Zhou et al., 2006) (Figure 2A). IRE1^LD^ mutants with truncations in the tail region are reported to associate with less BiP than full-length IRE1^LD^ do and, though regulated, have higher constitutive activity when overexpressed in ΔIRE1 cells, leading to the suggestion that BiP binds to the IRE1^LD^ tail region to repress IRE1 signaling (Oikawa et al., 2009). However, in *ΔERdj4* cells, ERdj4 was still able to increase BiP recovery with IRE1^CLD^-GST (Figure 2F), indicating its ability to act on the CLD.

BiP has been reported to associate with IRE1^LD^ in a nucleotide-independent manner via its nucleotide-binding domain rather than conventionally, by its substrate-binding domain (Carrara et al., 2015b). However, addition of ATP destabilized both the BiP-IRE1^LD^-GST complex and the endogenous BiP-IRE1 complex (Figures 2G and 2H). Together, these findings indicate that BiP engages the core structured region of IRE1^LD^ as a canonical Hsp70 substrate, an event that can be promoted by ERdj4.

### ERdj4 Opposes IRE1 Luminal Domain Dimerization in Cells and *In Vitro*

**Figure 3:**
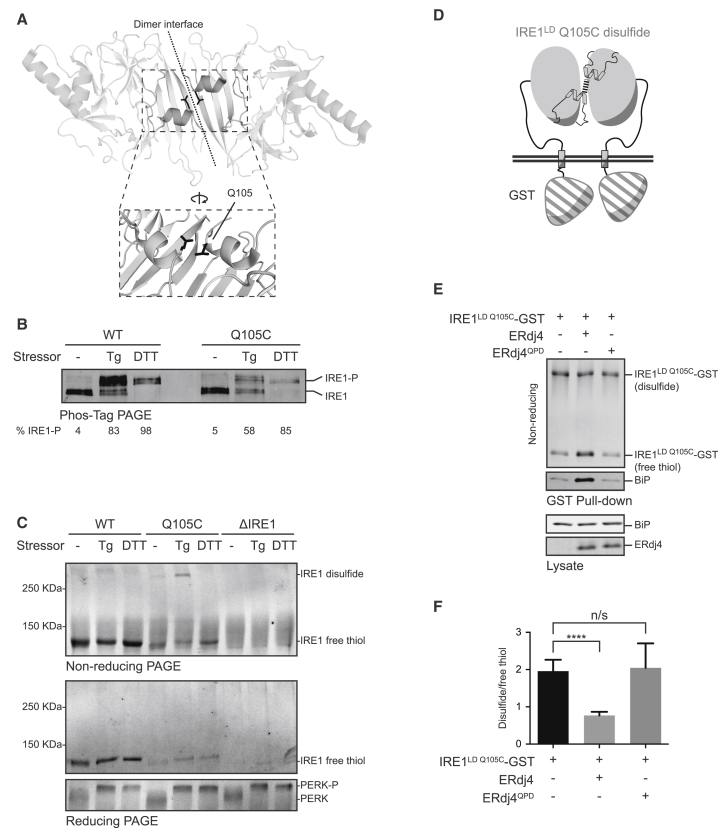
ERdj4 Opposes IRE1 Dimerization (A) Crystal structure of human IRE1^LD^ (PDB: 2HZ6) highlighting Q105 (black) at the dimer interface. (B) Reducing Phos-tag SDS-PAGE of endogenous IRE1α recovered from WT or IRE1^Q105C^ cells treated in the indicated manner. Fraction of active (phosphorylated) IRE1-P from this representative immuno blot is noted. (C) Representative immunoblot of endogenous IRE1α and PERK recovered from the indicated cell lines by immunoprecipitation of IRE1α or PERK and resolved by reducing and non-reducing SDS-PAGE. ER stress was induced by thapsigargin (Tg) or DTT. (D) Schema of IRE1^LD Q105C^-GST with the Q105C-Q105C disulfide indicated. (E) Representative immunoblot of IRE1^LD Q105C^-GST and BiP recovered from ΔERdj4 cells transfected with indicated constructs and resolved by non-reducing SDS-PAGE. (F) Ratio of disulfide-bound IRE1^LD^ Q105C-GST dimer to free thiol in indicated samples. Quantified in six independent experiments as shown in (D) above (mean ± SD, n = 6, ^∗∗∗∗^p < 0.0001, unpaired Student’s t test with Welch’s correction). See also Figure S2.

**Figure S2:**
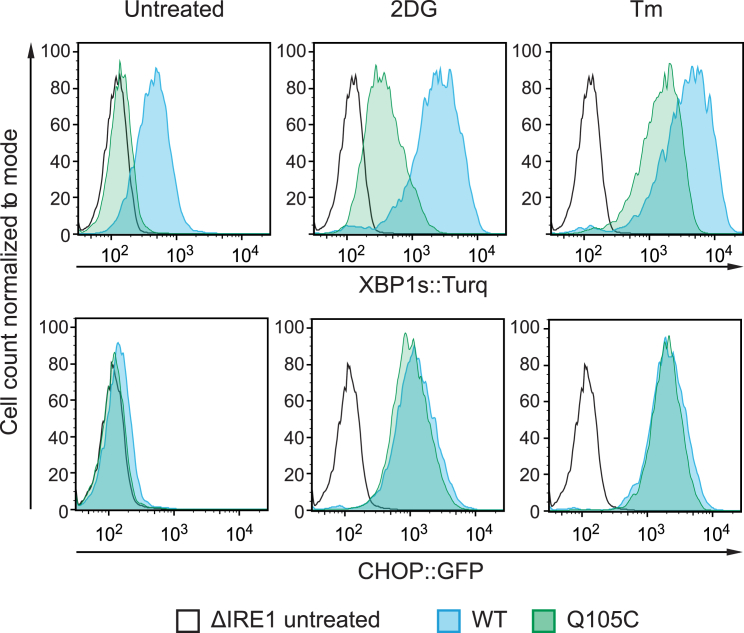
*ERN1*^*Q105C*^/*IRE1*^*Q105C*^ Encodes a Functional (albeit Attenuated) IRE1, Related to Figure 3 Histogram of XBP1s::Turquoise and CHOP::GFP signals obtained by flow cytometry analysis of the indicated cell lines untreated or exposed overnight to 2-deoxyglucose (2DG, 4mM), or tunicamycin (Tm, 2.5 μg/ml).

To determine whether the ERdj4-promoted BiP-IRE1^LD^ complex influenced the IRE1^LD^ monomer-dimer transition that initiates the UPR, we sought to establish a method to measure endogenous IRE1^LD^ dimerization in cells. The crystal structure of dimeric, active human IRE1^LD^ reveals a polar interaction between the side chains of Q105 from opposing protomers (Figure 3A, PDB: 2HZ6). Using CRISPR-Cas9-mediated homologous recombination, we replaced this residue with a cysteine at the endogenous *Ern1*/*Ire1α* locus. IRE1^Q105C^ CHO cells retained the ability to mount a UPR (Figures 3B and S2). IRE1^Q105C^ is expressed at a lower level than WT IRE1, possibly because it is less stable in cells. This decreased expression level likely accounts for the attenuated induction of the IRE1 branch of the UPR in IRE1^Q105C^ cells (Figure S2). Despite its lower level of expression, ER stress induction by thapsigargin-mediated luminal calcium depletion effected formation of a disulfide in the IRE1^Q105C^ mutant cells (Figure 3C), reflecting the close proximity of the cysteines in the activated dimer and providing a readout for stress-relevant IRE1^LD^ dimer formation *in vivo*.

A modified version of IRE1^LD^-GST with the Q105C mutation was used to gauge the effect of ERdj4 on the monomer-dimer ratio (Figure 3D). Unlike endogenous IRE1^Q105C^, exogenously expressed IRE1^LD Q105C^-GST is abundant and spontaneously forms disulfide-linked IRE1^LD Q105C^-GST dimers (Figure 3E). When co-transfected, WT ERdj4 decreased by 2.5-fold the fraction of disulfide-linked, dimeric IRE1^LD Q105C^-GST. ERdj4^QPD^ had no effect on the monomer-to-dimer ratio (Figures 3E and 3F).

**Figure 4:**
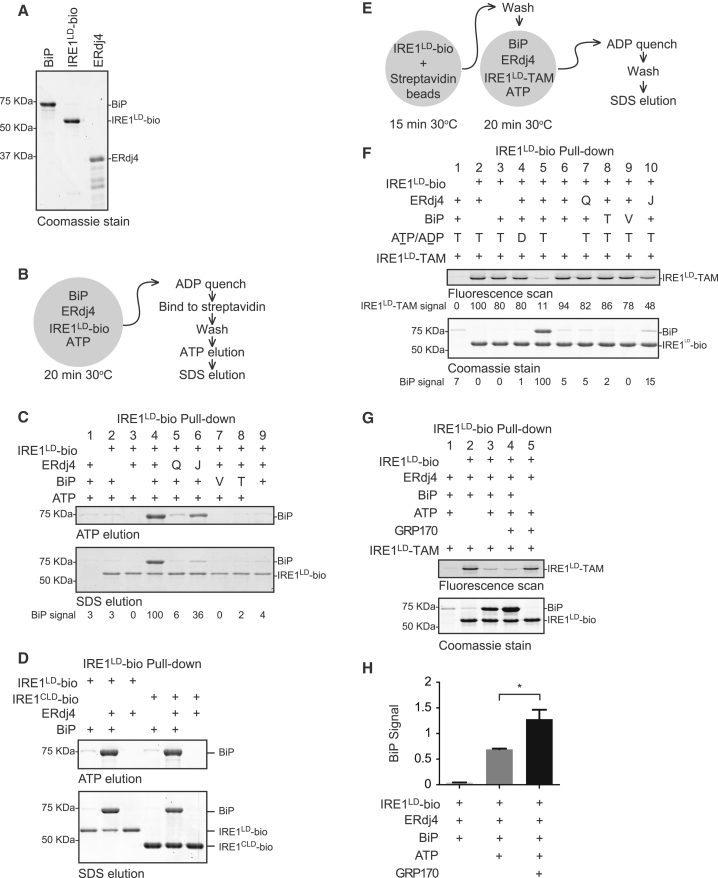
Complex Formation between BiP and IRE1 Requires ERdj4 (A) Coomassie stain of purified BiP, IRE1^LD^-bio, and ERdj4 resolved by SDS-PAGE. (B) Schema of the experiment shown in (C). (C) Coomassie-stained SDS-PAGE gel of biotinylated IRE1^LD^-bio and BiP recovered on a streptavidin matrix from reactions constituted as indicated. Concentrations used were 5 μM IRE1^LD^-bio, 8 μM ERdj4, 30 μM BiP, and 2 mM ATP. Q = ERdj4^QPD^, T = BiP^T229A^, V = BiP^V461F^, and J = isolated J domain of ERdj4. Proteins were eluted sequentially with ATP (ATP elution) and SDS sample buffer (SDS elution). (D) As in (B) and (C), with IRE1^CLD^. (E) Schema of the experiment shown in (F). (F) Sequential fluorescence scan and Coomassie stain of the same SDS-PAGE gel of proteins recovered on immobilized streptavidin from reactions assembled from the indicated components. The IRE1^LD^-bio-loaded beads were allowed to associate with fluorescently labeled IRE1^LD^-TAM, whose recovery in the pull-down reports on the integrity of the IRE1^LD^ dimer. Concentrations used were 0.5 μM IRE1^LD^-TAM, 8 μM ERdj4, 30 μM BiP, and 2 mM ATP. Q = ERdj4^QPD^, T = BiP^T229A^, V = BiP^V461F^, and J = isolated J domain of ERdj4. (G) As in (E) and (F), with 1 μM GRP170. (H) Quantification of the effect of GRP170 on BiP association with IRE1^LD^-bio, as in (G). Mean ± SD, n = 3, ^∗^p = 0.0223 by Student’s paired ratio t test. See also Figure S3.

**Figure S3:**
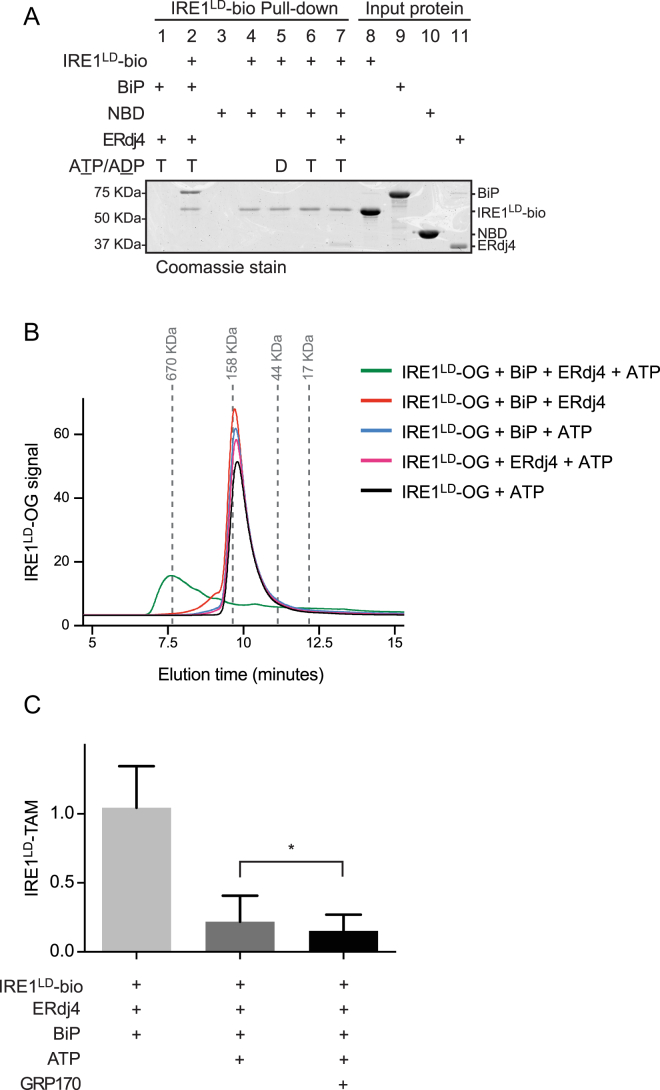
Canonical Complex Formation between BiP and IRE1, Related to Figure 4 (A) Coomassie-stained SDS-PAGE gel of biotinylated IRE1^LD^-bio and BiP recovered on a streptavidin matrix from reactions constituted as in Figure 4B, with BiP or the nucleotide-binding domain of BiP (NBD) as indicated. Note: We have not been able to observe noncanonical complex formation between IRE1^LD^ and the BiP NBD. (B) Fluorescence trace (Ex: 496 nm Em: 524 nm) of IRE1^LD^-OG elution from a Sec3 size-exclusion chromatography column. Reaction mixtures of the indicated composition were incubated at 30°C for 20 minutes and clarified at 21,000 *g* for 5 minutes. (C) Quantification of the effect of GRP170 on IRE1^LD^-TAM association with IRE1^LD^-bio, as in Figure 4G. Mean ± SD, n = 3, ^∗^p = 0.0293 by Student’s paired ratio t test.

To determine whether ERdj4 promoted the BiP-IRE1^LD^ complex by directly recruiting BiP to IRE1^LD^, we purified the three proteins from bacteria (Figure 4A). IRE1^LD^ was tagged with biotin on its C terminus (IRE1^LD^-bio). Formation of a BiP-IRE1^LD^-bio complex was assessed by recovery on immobilized streptavidin (Figure 4B). BiP and IRE1^LD^ formed a complex only in the presence of ERdj4 and ATP (Figure 4C, lanes 2, 4, and 9). Like its *in vivo* counterpart (Figures 2G and 2H), the isolated BiP-IRE1^LD^ complex thus formed was sensitive to disruption by incubation with ATP (Figure 4C, upper panel; the residual BiP eluted with SDS, lower panel, reflects incompleteness of the preceding ATP elution). The ERdj4^QPD^ mutation and mutations in BiP that interfered with its ATPase activity (BiP^T229A^) or substrate binding (BiP^V461F^) greatly enfeebled complex formation (Figure 4C, lanes 5, 7, and 8). Association of BiP’s isolated nucleotide-binding domain (NBD) with the IRE1^LD^ was not observed (Figure S3A), pointing away from the non-canonical IRE1-BiP interaction (previously suggested by Carrara et al., 2015b) and favoring instead formation of a complex in which BiP recognizes IRE1^LD^ as a typical substrate.

The isolated J domain of ERdj4 retained some ability to promote a BiP-IRE1^LD^ complex (Figure 4C, lane 6) but was reproducibly weaker than full-length ERdj4, attesting to the importance of the C-terminal targeting domain of ERdj4. It is likely that multiple BiP molecules engaged the IRE1^LD^-bio via multiple binding sites or as BiP oligomers (Figure S3B). In the presence of ATP, ERdj4 also recruited BiP to the biotinylated core IRE1^CLD^, indicating that the IRE1^LD^ tail region is not essential for complex formation between IRE1 and BiP (Figure 4D).

To determine whether ERdj4 and BiP interfere with IRE1^LD^ dimerization *in vitro*, we produced a non-biotinylated, tetramethylrhodamine-5-maleimide (TAMRA)-labeled fluorescent IRE1^LD^ probe (IRE1^LD^-TAM) and measured the effect of BiP, ERdj4, and ATP on recovery of IRE1^LD^-TAM in complex with IRE1^LD^-bio (Figure 4E). Consistent with the high affinity of IRE1^LD^ protomers for each other, IRE1^LD^-TAM formed a stable complex with IRE1^LD^-bio that was readily recovered on immobilized streptavidin. However, introduction of WT BiP, WT ERdj4, and ATP interfered with the IRE1^LD^ dimer while forming a BiP-IRE1^LD^-bio complex (Figure 4F). Completion of the BiP cycle by addition of the nucleotide exchange factor GRP170/ORP150 significantly increased the amount of BiP recovered with IRE1^LD^-bio and further attenuated recovery of IRE1^LD^-TAM (Figures 4G, 4H, and S3C).

**Figure 5:**
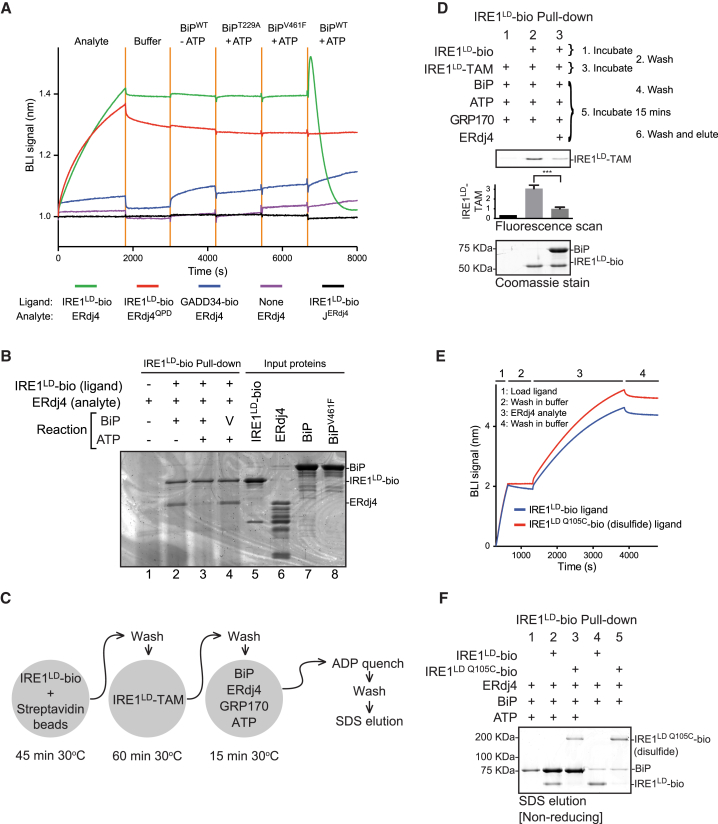
ERdj4 Recruits BiP to Disrupt IRE1 Dimerization (A) Bio-layer interferometry (BLI) signal of streptavidin sensors loaded with the indicated biotinylated ligand and reacted sequentially with the indicated solution of analyte, followed sequentially by the indicated solutions of BiP and ATP. Concentrations used were 1.5 μM ERdj4, 1 μM BiP, and 2 mM ATP. (B) Protein recovered from a BLI sensor lacking (lane 1) or containing an IRE1^LD^-bio ligand (lanes 2–4). The sensor was incubated with an ERdj4 analyte and then with BiP or BiP^V461F^ ± ATP. (C) Schema of the experiment shown in (D). (D) Fluorescence scans and Coomassie-stained SDS-PAGE gel of proteins recovered on immobilized streptavidin from reactions assembled from the indicated components. The IRE1^LD^-bio-loaded streptavidin beads were pre-associated with IRE1^LD^-TAM and then incubated in a solution of BiP, ERdj4, GRP170, and ATP. Bars show mean IRE1^LD^-TAM signal recovered with IRE1^LD^-bio (± SD) from four independent experiments, ^∗∗∗^p = 0.001 by parametric student’s paired ratio t test. (E) Time-dependent changes in BLI signal of sensors loaded with either WT biotinylated IRE1^LD^ (blue trace) or covalent dimeric disulfide-linked IRE1^LD Q105C^-bio (red trace) ligands. Ligand loading (step 1), wash (step 2), interaction with ERdj4 (step 3), and wash (step 4) are shown. (F) Coomassie stained non-reducing SDS-PAGE gel of IRE1^LD^-bio and BiP recovered on a streptavidin matrix from reactions constituted as in Figures 4B and 4C but with IRE1^LD^-bio or covalent dimeric disulfide-linked IRE1^LD Q105C^-bio. Proteins were eluted with SDS sample buffer. See also Figure S4.

**Figure S4:**
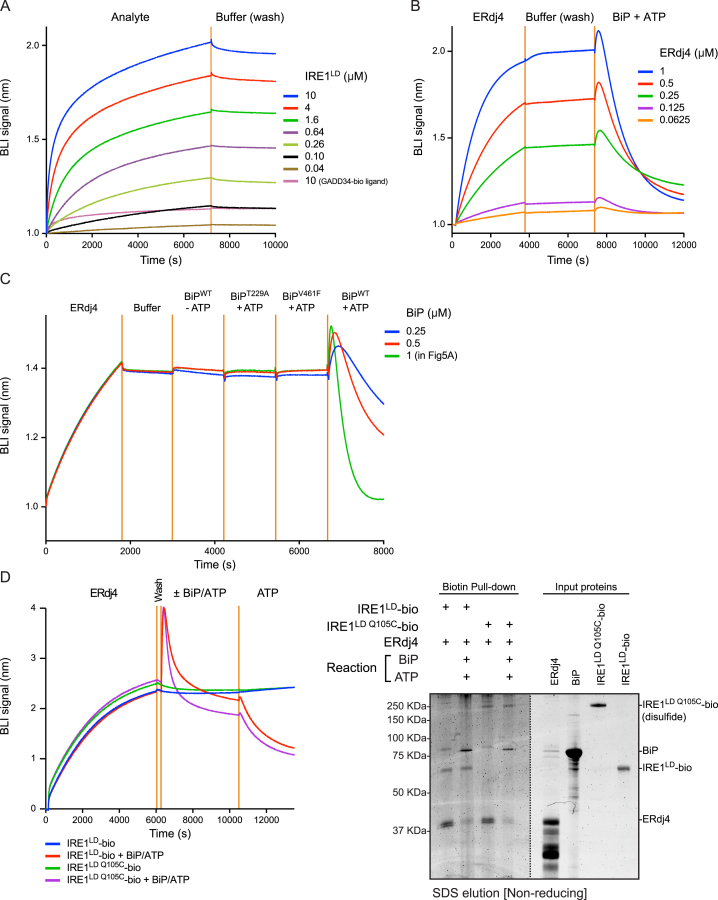
Disruption of Pre-formed IRE1^LD^-Dimers, Related to Figure 5 (A) Bio-layer interferometry (BLI) signal from streptavidin sensors pre-loaded with a biotinylated ERdj4 ligand (or with an irrelevant control biotinylated GADD34 ligand) and reacted with the indicated concentration of IRE1^LD^ as an analyte and then transferred to a buffer only (wash) solution. (B) BLI signal from streptavidin sensors pre-loaded with biotinylated IRE1^LD^ ligand and reacted with the indicated concentrations of ERdj4 as an analyte and transferred to a buffer only (wash) solution before incubation with 1 μM BiP and 2 mM ATP. (C) BLI signal from streptavidin sensors pre-loaded with biotinylated IRE1^LD^ ligand and reacted with ERdj4 as an analyte and then transferred to a buffer only (wash) solution before incubation with the indicated concentrations of BiP and 2 mM ATP. (D) (Left) BLI signal of streptavidin sensors loaded with the wild-type biotinylated IRE1^LD^, or covalent dimeric disulfide-linked biotinylated IRE1^LD Q105C^ ligands and reacted with ERdj4, followed sequentially by the indicated solutions. Concentrations used were 1.7 μM ERdj4, 6 μM BiP, 2 mM ATP. (Right) Coomasie-stained non-reducing SDS-PAGE gel of protein recovered by SDS sample buffer elution from the BLI sensors used (left). The dotted line indicates the boundary at which the image contrast/brightness properties were treated differently to make the image clearer. Note: To enable formation of Q105C-disulfide, without interference by other cysteines, both the WT IRE1^LD^ and the IRE1^LD Q105C^ ligands were surface biotinylated on exposed lysine residues. This coupling chemistry likely accounts for the differences in kinetics of the BLI signal observed in this experiment as compared with (A), (B), and (C) and Figure 5A, in which the IRE1^LD^ ligand was biotinylated on a single C-terminal cysteine residue (D443C) using maleimide biotin

Bio-layer interferometry (BLI) was used to dissect ERdj4-mediated BiP recruitment to IRE1^LD^. IRE1^LD^-bio immobilized onto the BLI sensor readily associated with both WT ERdj4 and mutant ERdj4^QPD^, generating a robust BLI signal (Figure 5A). The isolated J domain of ERdj4 did not interact with the IRE1^LD^-bio sensor, implicating the C-terminal domain of ERdj4 in the interaction. In the absence of ATP, the binary complex of IRE1^LD^-bio and ERdj4 (stabilized by the very low *k*_off_ rate of the complex, Figure S4A) interacted minimally with BiP and did not interact with mutants of BiP (BiP^T229A^ and BiP^V461F^), even in the presence of ATP. However, immersing the sensor loaded with the IRE1^LD^-bio-ERdj4 complex into a solution of BiP and ATP gave rise to a highly reproducible, transient, positive BLI signal, followed by its decline toward the baseline signal of the IRE1^LD^-bio-loaded BLI sensor (observed before formation of the IRE1^LD^-bio-ERdj4 complex; Figure 5A, green trace). The kinetics of this biphasic swing in the BLI signal were increased both by the amount of ERdj4 bound to the IRE1^LD^-bio-loaded BLI sensor (Figure S4B) and by the concentration of BiP (Figure S4C).

Analysis of the protein content of the BLI sensor preceding and following its immersion into the solution containing BiP and ATP revealed the presence of ERdj4 in the former steady state and its absence from the latter (Figure 5B). These observations are consistent with ERdj4’s ability to promote formation of a complex between BiP and IRE1^LD^ through directed ATP hydrolysis and to maintain this complex by facilitating BiP re-binding following nucleotide exchange. BiP binding disrupts the otherwise stable IRE1^LD^-ERdj4 complex. When ERdj4 is present at adequate concentration, its re-binding to IRE1^LD^ dynamically maintains the IRE1^LD^-BiP complex (Figure 4C). However, ERdj4 dissociated from the BLI sensor is too dilute to rebind, allowing the IRE1^LD^-BiP complex to dissipate through nucleotide exchange (last segment of green trace in Figure 5A).

To determine whether ERdj4 and BiP could forcibly disrupt pre-formed IRE1^LD^ dimers or merely repress IRE1^LD^ dimerization by retaining monomers, we confronted pre-formed IRE1^LD^-bio/IRE1^LD^-TAM dimers, immobilized on streptavidin, with BiP, GRP170, and ATP in the presence or absence of ERdj4 and monitored the loss of bound IRE1^LD^-TAM (Figure 5C). Dissociated IRE1^LD^-TAM was diluted in the large assay volume, minimizing the effect of rebinding. ERdj4 accelerated the loss of IRE1^LD^-TAM (Figure 5D), indicating that ERdj4 empowered BiP to split pre-existing IRE1 dimers.

To determine if ERdj4 could recruit BiP to dimeric IRE1^LD^ (a prerequisite for the forceful disruption suggested by Figure 5D), biotinylated IRE1^LD Q105C^ was purified and allowed to form Q105C-Q105C disulfides, covalently stabilizing the biotinylated IRE1^LD Q105C^ dimers. ERdj4 bound (Figure 5E) and recruited BiP to disulfide-linked dimeric biotinylated IRE1^LD Q105C^ (Figure 5F), suggesting the existence of an allosteric component to BiP-mediated inhibition of IRE1. BiP and ATP were also able to remove ERdj4 bound to the biotinylated disulfide-linked dimeric biotinylated IRE1^LD Q105C^, indicating that IRE1 de-dimerization is not a strict prerequisite for BiP-mediated ERdj4 displacement (Figure S4D).

### Unfolded Protein Substrates Compete with IRE^LD^ for BiP, Restoring IRE1^LD^ Dimers

**Figure 6:**
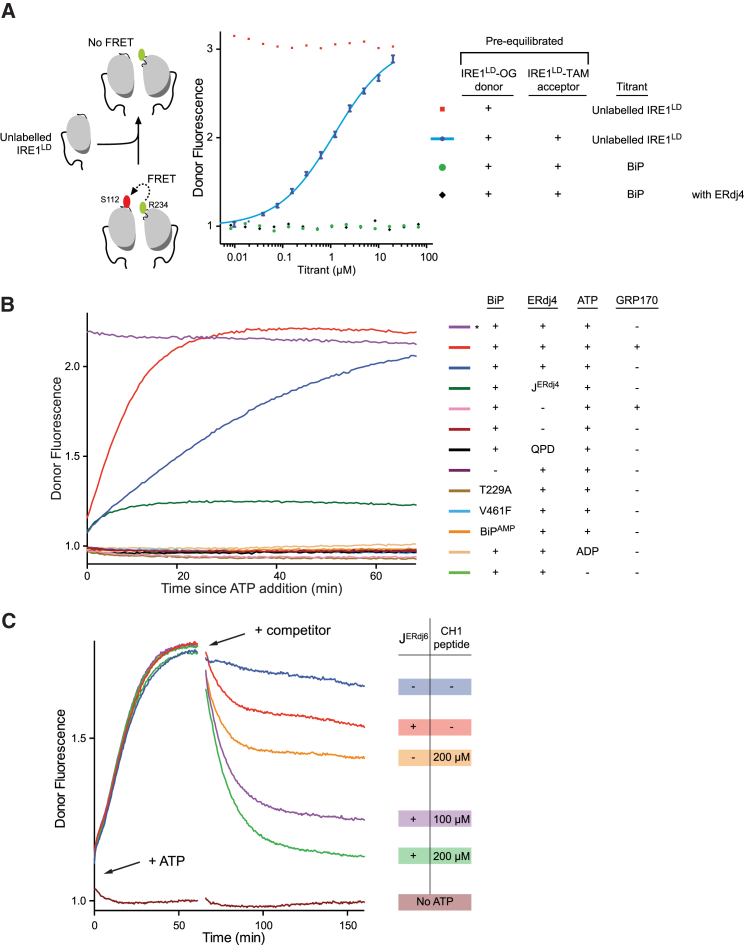
Unfolded Proteins Compete for BiP to Restore the IRE1 Dimer (A) Donor fluorescence as a function of the concentration of competing unlabeled IRE1^LD^ equilibrated with a FRET pair (0.2 μM labeled IRE1^LD^) consisting of an IRE1^LD^-OG^488^ donor (conjugated at R234C) and IRE1^LD^-TAM acceptor (conjugated at S112C) (blue trace, mean values ± SD from three independent experiments). Also shown are titrations of unlabeled IRE1^LD^ into a mock FRET sensor (no IRE1^LD^-TAM acceptor; red trace) and titration of BiP with ADP (± ERdj4) into the pre-equilibrated FRET pair (green and black traces). (B) Time-dependent change in donor fluorescence of the IRE1^LD^ FRET pair from (A) incubated at t = 0 with the components shown to the right. Concentrations used were 0.2 μM FRET IRE1^LD^, 30 μM BIP, 2.5 μM ERdj4, 1 μM GRP170, and 2 mM ATP. J^ERdj4^ lacks the C-terminal targeting region. BiP^AMP^ is AMPylated BiP. The asterisks marks a reaction set up with a mock FRET sensor lacking the IRE1^LD^-TAM acceptor. (C) Time-dependent change in donor fluorescence of the IRE1^LD^ FRET pair exposed at t = 0 to BiP, ERdj4 and ATP (arrow labeled “+ ATP”). Concentrations used were 0.2 μM FRET IRE1^LD^, 50 μM BIP, 2.5 μM ERdj4, and 2 mM ATP. Following disruption of the FRET pair, at 60 min, the sample was injected with BiP binding peptide and the J domain of ERdj6 (2.5 μM) (arrow labeled “+ competitor”). See also Figure S5.

To monitor the disruption of IRE1^LD^ dimers in real time, we developed a Förster resonance energy transfer (FRET)-based assay that reports on the IRE1^LD^ monomer-dimer equilibrium. IRE1^LD^ molecules labeled on a single cysteine introduced at R234 with an Oregon green 488 donor were combined with molecules labeled on a single cysteine introduced at S112 with a TAMRA acceptor. A robust FRET signal (predicted by proximity of R234 and S112 in the human IRE1^LD^ dimer crystal structure, PDB: 2HZ6), reflected by the quenching of donor fluorescence, was detected. Addition of unlabeled IRE1^LD^ restored donor fluorescence nearly to levels observed in absence of acceptor molecules, confirming the role of dimerization in the quenched donor fluorescence (Figure 6A).

**Figure S5:**
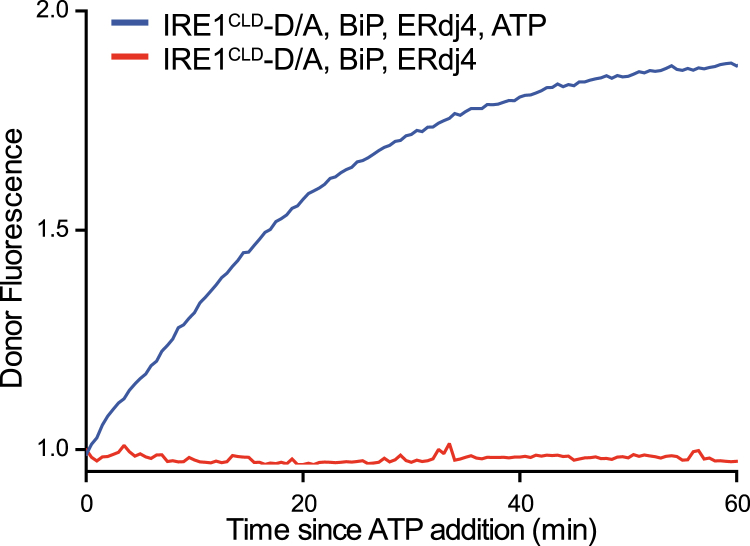
ERdj4 and BiP Monomerize the IRE1 Core Luminal Domain, Related to Figure 6 As in Figure 6B, but with OG488 and TAMRA-labeled IRE1^CLD^.

Extended incubation of donor- and acceptor-labeled IRE1^LD^ molecules with high concentrations of ADP-bound BiP (in the absence or presence of ERdj4) did not disrupt the FRET signal, indicating that IRE1^LD^ is a poor equilibrium BiP substrate (Figure 6A, lower traces). However, addition of BiP, ERdj4, and ATP to pre-equilibrated donor and acceptor IRE1^LD^ reversed the FRET such that donor fluorescence nearly equaled that observed in the absence of acceptor molecules. BiP mutants defective in ATP hydrolysis (BiP^T229A^) and substrate binding (BiP^V461F^) and ERdj4^QPD^ were inert, as was BiP^AMP^ that had been inactivated by AMPylation (Preissler et al., 2015b). The isolated J domain of ERdj4 could not drive efficient monomerization. Addition of GRP170 significantly increased the rate of IRE1^LD^ monomerization (Figure 6B). BiP, ERdj4, and ATP also disrupted the FRET observed between donor and acceptor labeled IRE1 CLD (IRE1^CLD^, Figure S5).

Addition of a BiP substrate (CH1 peptide) (Marcinowski et al., 2011) alone weakly restored the FRET signal to samples maintained in the monomeric state by ERdj4, BiP, and ATP. However, introduction of sub-stoichiometric amounts of a second J-protein (devoid of IRE1^LD^-binding activity), alongside the BiP substrate, markedly accelerated re-formation of the FRET signal (Figure 6C). These observations suggest that BiP binding to a substrate peptide directed by an orthogonal J-protein can compete successfully for ERdj4-directed, BiP-mediated IRE1^LD^ monomerization. The transitions between the monomeric “low-FRET” and dimeric “high-FRET” states (Figure 6C) occur on a 30- to 60-min timescale similar to that of the dissolution of the BiP-IRE1 complex in stressed cells and its reformation in cells recovering from stress (Bertolotti et al., 2000; Figure 4 therein), suggesting that IRE1^LD^, BiP, J-proteins, and a BiP substrate together can recapitulate in a simple *in vitro* assay, a key aspect of UPR signaling.

## Discussion

The discovery of an ER-localized J-protein that selectively represses IRE1 activity has paved the way for experimental reconstitution of a UPR that is based on sensor repression by free chaperone and de-repression by accumulating unfolded proteins. The experiments incorporating this missing component have thus closed an important gap between the finding that activity of the UPR transducers in cells correlates inversely with the amount of associated BiP and a plausible model for how this might come about.

The structural basis for the disruption of dimeric IRE1^LD^ by ERdj4-recruited BiP remains to be determined. However, ERdj4’s ability to recruit BiP to disulfide-stabilized IRE1^LD^ dimers *in vitro* suggests that chaperone-mediated IRE1 monomerization may proceed through an unstable tertiary IRE1_2_-BiP intermediate and that BiP interferes with IRE1 dimerization by allosterically disrupting the dimer interface, not merely by binding to and blocking the dimer interface. Such a process, dependent on energy released by ERdj4-promoted ATP hydrolysis by BiP, may be analogous to auxilin-directed, Hsc70-mediated destabilization of clathrin coats (Xing et al., 2010) or DnaJ-directed, DnaK-mediated destabilization of *E. coli* σ^32^ (Rodriguez et al., 2008).

In eukaryotic cells, BiP is highly abundant, whereas IRE1 is scarce (Ghaemmaghami et al., 2003, Kim et al., 2014). Given this stoichiometry, how might BiP repression be reconciled with the high sensitivity of the UPR that is observed empirically (Pincus et al., 2010)? Alone, BiP has very low equilibrium affinity for IRE1, likely a reflection of the lack of accessible high-affinity BiP binding site(s) on the regulatory core of IRE1^LD^. Instead, the BiP-IRE1 complex is maintained through a dynamic, non-equilibrium, ATP-consuming process of J-protein-driven cycles of BiP rebinding to IRE1^LD^ and release following nucleotide exchange. The ability of the nucleotide exchange factor GRP170 to increase both the amount of BiP recruited to IRE1^LD^ and the rate of IRE1^LD^ monomerization *in vitro* points to the importance of the ATP-bound pool of BiP to IRE1 repression, as it is best explained by GRP170’s ability to recover BiP molecules that have futilely hydrolyzed ATP without recruitment onto IRE1^LD^, thereby increasing the concentration of ATP-bound BiP.

**Figure 7:**
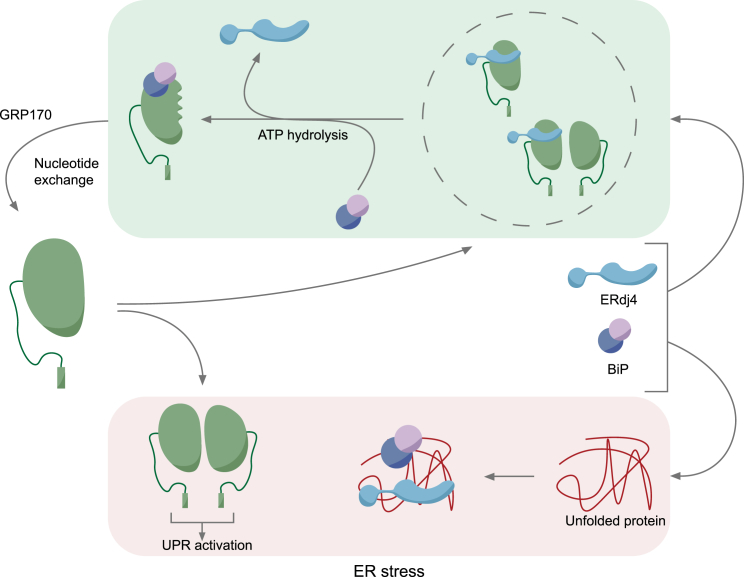
IRE1 Repression by ERdj4 and BiP and Activation by Unfolded Proteins In the unstressed ER (green shading), ERdj4 binds the IRE1 CLD via its C-terminal targeting domain. ERdj4 stimulates BiP’s ATPase activity to promote BiP binding to IRE1, ejection of ERdj4, and formation of a repressive BiP-IRE1 complex with a disrupted dimer interface. The BiP-IRE1 complex turns over by nucleotide exchange. Free ERdj4 and BiP recruit the released IRE1 (either as a monomer or dimer) in a kinetically maintained repressive cycle. Accumulating unfolded proteins during ER stress (red shading) compete for BiP and/or ERdj4, interrupting the cycle of repression. IRE1 monomers are free to dimerize and activate downstream signals.

A repressive complex maintained dynamically by J-accessible, substrate-free, and ATP-bound BiP molecules would be sensitive to the concentration of unfolded proteins, as these compete for the same pool of BiP (Figure 7). It is noteworthy that analysis of cell lysates suggests that the pool of BiP available for IRE1 repression is small: most of the BiP detectable on native gels is either substrate bound, engaged in inactive BiP oligomers, or inactivated by AMPylation (Freiden et al., 1992, Preissler et al., 2015a, Preissler et al., 2015b). Thus, the buffer of ATP-bound BiP available to repress IRE1 is likely rather modest.

The strict requirement for a J-protein for repressive complex formation suggests that competition may also occur at the level of the co-chaperone. ERdj4, far less abundant than BiP, likely possesses affinity for certain unfolded proteins through its C-terminal targeting domain (Dong et al., 2008, Shen et al., 2002). Since ERdj4 is similarly dependent on this domain to repress IRE1 both *in vitro* and *in vivo*, it follows that the canonical chaperone-substrate interaction underlying IRE1 repression could be out-competed by ERdj4 ligands and serve as selective activators of the IRE1 branch of the UPR.

There is nothing in our findings to argue against an additional role for unfolded proteins as activating IRE1 ligands. However, ERdj4-directed BiP repression is played out at the level of the minimal structured core IRE1^LD^, sufficient for UPR regulation (Oikawa et al., 2009). In pure solution, core IRE1^LD^ protomers have a high affinity for each other and dimerize without a stabilizing ligand (Zhou et al., 2006). Furthermore, the formation of a stress-dependent IRE1^LD Q105C^ disulfide is difficult to reconcile with an extended unfolded protein engaging the proposed peptide binding groove of IRE1^LD^ as the initiator of IRE1^LD^ dimerization. If unfolded proteins contribute to IRE1 activity, it seems they do so not by altering the monomer-dimer equilibrium, the crucial, first, regulatory step of IRE1 signaling, but rather by influencing the formation of higher-order IRE1 oligomers (Karagöz et al., 2017).

A J-protein that selectively represses IRE1 and is, in turn, a selective transcriptional target of XBP1 (Adamson et al., 2016, Lee et al., 2003) constitutes a strand-specific negative-feedback loop. The intriguing parallels in phenotype between XBP1 overexpression and ERdj4 deletion, as reflected in defective B cell development (Fritz and Weaver, 2014), suggest an important premium attached to selectively capping the expression of XBP1 target genes. To attain this selectivity, ERdj4 likely exploits structural differences between PERK^LD^ and IRE1^LD^. This should not be difficult, given that these proteins attain their homologous structure via very limited sequence identity.

ERdj4 deletion only partially deregulates IRE1. Residual IRE1-BiP complexes found in *Δ*ERdj4 cells are likely catalyzed by other ERdjs, which may also target BiP to PERK^LD^. ERdj2 is a plausible candidate and may play a role in repressing both the IRE1 and PERK branches of the UPR. This remains a speculation, as the experiments performed here do not distinguish between a role in protein-folding homeostasis from direct recruitment of BiP to the luminal domains of the UPR transducers. Indeed, it remains possible that several ERdj proteins contribute to both processes, accounting for the observed redundancy in their function.

Recent work has emphasized the importance of the translocon in IRE1 regulation (Adamson et al., 2016), with evidence that disruption of a complex involving Sec61, ERdj2/Sec63, and IRE1 deregulates IRE1 signaling (Plumb et al., 2015, Sundaram et al., 2017). Thus, given the evidence uncovered here for the critical role of J-proteins in catalyzing a repressive BiP-IRE1 complex, it is tempting to propose that translocon-associated ERdj2 may couple IRE1 signaling directly to the flux of proteins imported into the ER. When the flux is low, ERdj2 recruits BiP to IRE1^LD^. During periods of high secretory activity, ERdj2 is engaged in facilitating translocation, thereby allowing IRE1 to dimerize and activate. Through this coupling, the UPR may activate even before unfolded proteins begin to accumulate in the ER.

By exploiting the diversity of functionalities associated with J-proteins, BiP-mediated repression of ER stress transducers emerges as a conserved, sensitive, and potentially versatile mechanism for coupling changes in unfolded protein burden to signaling—the essence of the UPR.

## STAR★Methods

### Key Resources Table

**Table:** 

REAGENT or RESOURCE	SOURCE	IDENTIFIER
**Antibodies**		

Polyclonal Rabbit anti-mouseIRE1α	Bertolotti et al., 2000	NY200
Polyclonal Chicken anti-hamsterBiP	Avezov et al., 2013	Anti-BiP
FLAG-M2	Sigma	Cat no: F1804 RRID:AB_262044
Polyclonal Rabbit anti-GST	Ron and Habener, 1992	Anti-CHOP
Polyclonal Rabbit anti-mousePERK-P	Bertolotti et al., 2000	NY97
Polyclonal Rabbit anti-mousePERK	Bertolotti et al., 2000	NY201

**Bacterial and Virus Strains**		

BL21 C3013 *E. coli*	NEB	Cat no: C3013I
Origami B(DE3) *E. coli*	Novagen/MERCK	Cat no: 70837
**Chemicals, Peptides, and Recombinant Proteins**		

Thapsigargin	Calbiochem	Cat no: 586005
Tunicamycin	Melford	Cat no: T2250
2-Deoxyglucose	Sigma	Cat no: D6134
4μ8c	Tocris Bioscience	Cat no: 4479
Hexokinase	Sigma	Cat no: H4502
Phos-tag acrylamide	Distributed by Wako Pure Chemical Industries Synthesized by NARD institute	NARD: AAL-107 Wako cat: 300-93523
Digitonin	Calbiochem	Cat no: 300410
Biotin-maleimide	Sigma	Cat no: B1267
Oregon Green-iodoacetic acid	ThermoFisher	Cat no: O6010
TAMRA-maleimide	Sigma	Cat no: 94506
Biotin-NHS ester	Sigma	Cat no: H1759
Phosphocreatine	Sigma	Cat no: 10621714001
Biotin	Thermo Scientific	Cat no: B1595
Creatine kinase	Sigma	Cat no: C3755

**Experimental Models: Cell Lines**		

Clone S21 a derivative of RRID: CVCL_0214	Sekine et al., 2016	CHO S21

**Oligonucleotides**		

See Table S2 for Oligonucleotides	N/A	N/A

**Recombinant DNA**		

See Table S1 for Recombinant DNA	N/A	N/A

**Software and Algorithms**		

FlowJo,LLC,		RRID: SCR_008520
GraphPad-Prism V7		RRID: SCR_002798
ImageJ		RRID: SCR_003070

**Other**		

Octet RED96	Pall ForteBio	
1200 Infinity (HPLC)	Agilent technologies	
Aktapurifier (FPLC)	GE healthcare	
CLARIOstar platereader	BMG Labtech	

### Contact for Reagent and Resource Sharing

Further information and requests for resources and reagents should be directed to and will be fulfilled by the Lead Contact, David Ron (dr360@medschl.cam.ac.uk).

### Experimental Model and Subject Details

#### CHO cell line

A parental stock of Chinese Hamster Ovary CHO-K1 cells (ATCC, CCL-61) was used. Its identity has been validated by the presence of auxotrophic markers and by deep sequencing of the genome.

*CHOP::GFP* and *XBP1::Turquoise* reporters were introduced sequentially under G418 and puromycin selection to generate the previously-described derivative CHO-K1 S21 clone (Sekine et al., 2016). The puromycin resistance marker was subsequently lost, rendering CHO-K1 S21 cells sensitive to puromycin.

Adherent CHO cell-lines were grown in Ham’s nutrient mixture F12 (Sigma). All cell media was supplemented with 10% Hyclone FetalClone-2 serum (FetalClone II, Hyclone-GE Healthcare Life Sciences, South Logan, UT Lot# ABB214492), 2mM glutamine (Sigma), 100 U/ml penicillin and 100 μg/ml streptomycin (Sigma). Cells were grown at 37°C in 5% atmospheric CO_2_.

#### Bacterial culture

Proteins were expressed in BL21 C3013 *E. coli* cells or Origami B(DE3) cells (NEB). Bacterial cultures were grown at 37°C in LB medium containing 100 μg/ml ampicillin to an OD_600nm_ of 0.6-0.8. Expression was induced with 0.5 mM isopropylthio β-D-1-galactopyranoside (IPTG) and the cells were further incubated for 16 hours at 18°C.

### Method Details

#### Cell culture

Thapsigargin (Calbiochem) treatment was at 0.5 μM. Tunicamycin (Melford) treatment was at 2.5 μg/ml for 16 hours unless otherwise stated. 2-Deoxyglucose (Sigma) treatment was at 4 mM for 16 hours. Dithiothreitol (DTT) (Sigma) treatment was at 2 mM for 15 minutes. 4μ8c (Cross et al., 2012) treatment was at 10 μM for 12 days.

#### Transfection

Cells were transfected using Lipofectamine LTX (Life Technologies) with the reduced serum medium Opti-MEM (Life Technologies) according to manufacturer’s instructions.

#### Gene manipulation and allele analysis

Cas9 guide design was aided in part by the online resource “CRISPy” (Ronda et al., 2014) though several guides were designed manually following standard guidelines (Ran et al., 2013). Cells were transfected with the Cas9 and guide constructs and grown for a week before analysis by flow cytometry and sorting.

For genotyping, genomic DNA was extracted from cells by incubation in proteinase K solution (100 mM Tris pH 8.5, 5 mM EDTA, 200 mM NaCl, 0.25% SDS, 0.2 mg/ml Proteinase K) overnight at 50°C. Proteinase K was then heat inactivated at 98°C for 20 minutes before the supernatant was collected and used as a template in PCR reactions before sequencing. To aid in interpreting sequencing data of genes modified by Cas9 the changes in size of the target gene alleles were determined by capillary electrophoresis on a 3730xl DNA analyzer (Applied Biosystems). Samples to be analyzed by the DNA analyzer were generated through PCR reactions where one of the oligonucleotides had a 5′ 6-carboxyfluorescein (FAM) flurophore modification. Genomic information of the clones elaborated, is provided in supplemental table S3

##### Creating the endogenous IRE1^Q105C^

The endogenous IRE1 locus was challenged with Cas9 guide UK1558 to generate a loss of function indel and fluorescence-activated cell sorting (FACS) was used to select for XBP1s::Turquoise dull cells after 2-deoxyglucose (2DG) treatment. The resultant clones were genotyped with oligonucleotides 1100/1125 and 1101. After sequencing, a clone (NC6) that was apparently homozygous for a single frameshift nucleotide deletion was selected. To introduce the Q105C and C109S mutations, the new IRE1 locus was challenged with Cas9 guide UK1559 and a PCR-knitting generated repair template (oligonucleotides used: 1097, 1098, 1116, 1117 to PCR from CHO genomic DNA, see supplemental table S4 for repair template sequence). Cells that successfully repaired the IRE1 locus were selected using FACS to collect cells that were XBP1s::Turquoise bright after 2DG treatment. Resultant clones were genotyped with oligonucleotides 1100/1125 and 1101. Two clones (CV1 and CV8) were idenitfied by sequencing as homozygous for the repair sequence and were used for subsequent experiments.

#### Flow cytometry and FACS

Flow cytometry was carried out on a BD LSRFortessa. Adherent CHO cells were washed once in PBS and then incubated for 5 minutes in PBS + 4 mM EDTA before harvesting and fixing in PBS + 1.1% paraformaldehyde. Cell sorting was carried out on a Beckman Coulter MoFlo Cell sorter. Adherent CHO cells were washed once in PBS and then incubated 5 minutes in PBS + 4 mM EDTA + 0.5% BSA before sorting into fresh media. CHOP::GFP fluorescence was measured by excitation at 488 nm and monitoring emission at 530/30 nm. XBP1s::Turquoise fluorescence was measured by excitation 405 nm and monitoring emission 450/50 nm.

#### Mammalian cell lysis

Adherent cells were grown in 10-cm dishes and treated as described above. The dishes were then transferred to ice and cells were washed in PBS and harvested in PBS + 1 mM EDTA with a cell scraper. The collected cells were spun at 370 *g* for 5 minutes at 4°C. Cells were lysed in 1% Triton X-100, 150 mM NaCl, 20 mM HEPES-KOH pH 7.5, 10% glycerol, 1 mM EDTA, 1 mM phenylmethylsulphonyl fluoride (PMSF), 4 μg/ml Aprotinin, and 2 g/ml Pepstatin A and 2 μM Leupeptin. For BiP coimmunoprecipitation experiments the lysis buffer lacked EDTA and was further supplemented with 10 mM MgCl_2_, 6 mg/ml glucose and 2 mg/ml Hexokinase (Sigma) to deplete ATP and stabilize BiP substrate interactions. For analysis of IRE1 phosphorylation by Phos-tag gel electrophoresis (Yang et al., 2010), the lysis buffer was further supplemented with 10 mM tetrasodium pyrophosphate, 100 mM sodium fluoride and 17.5 mM β-glycerophosphate. For analysis of IRE1^Q105C^ disulfide linked species the lysis buffer was further supplemented with 20 mM N-Ethylmaleimide (NEM). After 5 minutes of lysis on ice, cells were spun at 21,130 *g* for 10 minutes at 4°C. The supernatant was transferred to a fresh tube and, when necessary, protein concentration measured with Bio-Rad protein assay (Bio-Rad).

To reduce the non-specific binding of BiP to protein-A Sepharose beads the experiments shown in Figures 1D, 1E, and 2H included an additional digitonin permeabilization step (Le Gall et al., 2004) to remove non-membrane associated BiP from cells prior to lysis. After harvesting, cells were washed in HNC buffer (50 mM HEPES-KOH pH 7.5, 150 mM NaCl, 2 mM CaCl_2_) and then incubated in HNC with 0.1% (w/v) digitonin (Calbiochem) for 10 minutes. Cells were then washed in HNE buffer (50 mM HEPES-KOH pH 7.5, 150 mM NaCl, 1 mM EGTA) before proceeding to the lysis step as described above.

#### Antibodies

Anti-mouse IRE1α serum (NY200) and anti-mouse PERK serum (NY97 & NY201) was used for immunoprecipitation and immunoblot detection of endogenous IRE1α, PERK and PERK-P respectively (Bertolotti et al., 2000). Anti-hamster BiP serum was used for immunoblot detection of endogenous BiP (Avezov et al., 2013). Anti-GST serum was used for immunoblot detection of GST fusion proteins (Ron and Habener, 1992). Anti-FLAG-M2 monoclonal antibody was used for immunoblot detection of FLAG fusion proteins (Sigma F1804).

#### Immunoprecipitation and GSH pull-down assays

Protein A Sepharose 4B beads (Zymed Invitrogen) and appropriate antisera (against IRE1 and PERK), or glutathione (GSH) Sepharose 4B beads (GE Healthcare) were equilibrated in lysis buffer. 20 μL beads per sample were added to lysates and left rotating for 1 hour at 4°C. The beads were then washed in lysis buffer and residual liquid was removed using a syringe. The protein from the beads was eluted in SDS sample buffer containing 20 mM DTT or 20 mM NEM (for non reducing gels).

#### SDS-PAGE/Phos-tag SDS-PAGE and immunoblotting

Samples were separated on standard polyacrylamide Tris-HCl gels and transferred to Immobilon-P PVDF membrane (Pore size 0.45 μm, Sigma). The membrane was then blocked in 5% (w/v) dried skimmed milk in PBS. For the non-reducing gels of endogenous IRE1α (recovered by immunoprecipitation) the membrane was treated with GDHCl buffer (6 M Guanidine-HCl, 250 mM NaCl, 50 mM Tris pH 7.5, 10% glycerol) with added 0.2% SDS and 100 mM DTT for 30 minutes then washed in GDHCl buffer, and finally treated with GDHCl buffer with added 40 mM NEM for 30 minutes. The membranes were then washed three times in TBS (50 mM Tris pH 7.5, 150 mM NaCl) and blocked in 5% (w/v) dried skimmed milk in PBS before continuing with the standard procedure. After blocking the membranes were washed in TBS with 0.1% Tween-20 and stained with various primary antibodies/antisera followed by staining with IRDye fluorescently labeled secondary antibodies or horse radish peroxidase (HRP) labeled secondary antibodies (G21234, ThermoFisher). Super Signal West Pico Chemiluminescent substrate (Thermo Scientific) was used as an HRP substrate. Imaging was carried out with either a LICOR CLx Odyssey infrared imager or by film. For Phos-tag gels, 50 μM Phos-tag acrylamide (NARD) and 100 μM MnCl_2_ were included in the gel recipe as described (Kinoshita et al., 2009). Transfer was carried out according to the standard protocol except that prior to transferring, the Phos-tag gel was washed in transfer buffer supplemented with 1 mM EDTA.

Coomassie-staining was carried out with Instant Blue (Expedeon) and imaged on the above-mentioned LICOR. IRE1^LD^-TAM in SDS-PAGE gels was imaged with a typhoon trio imager with a 532 nm laser and monitoring emission at 580/30 nm.

Signal quantitation from SDS-PAGE gels or from immunoblots was carried out using the ImageJ software (NIH).

#### Protein Purification

##### Human IRE1^LD^

IRE1^LD^ (UK2007), IRE1^LD^-cys (UK1915) and IRE1^CLD^-cys (UK1998) used to make IRE1^LD^-biotin (UK1915 and UK2007), IRE1^LD^-OG (UK1915) and IRE1^CLD^-biotin (UK1998), were encoded on pGEX vectors (GE Healthcare) as GST fusion proteins and expressed in BL21 C3013 *E. coli* cells (NEB). Bacterial cultures were grown at 37°C in LB medium containing 100 μg/ml ampicillin to an OD_600nm_ of 0.6-0.8. Expression was induced with 0.5 mM IPTG and the cells were incubated for 16 hours at 18°C. The cells were sedimented by centrifugation and the pellets were resuspended in TNGMT buffer (50 mM Tris pH 7.4, 500 mM NaCl, 10% glycerol, 1 mM MgCl_2_, 1 mM TCEP). The cell suspension was supplemented with 0.1 mg/ml DNaseI and protease inhibitors [2 mM PMSF, 4 μg/ml pepstatin, 4 μg/ml leupeptin, 8 μg/ml aprotinin] and lysed by repeated passage through a high-pressure homogenizer (EmulsiFlex-C3, Avestin). The lysates were cleared by centrifugation at 20,000 *g* for 60 minutes. The supernatant was removed, supplemented with 0.5% (v/v) Triton X-100, and incubated for 60 minutes at 4°C with glutathione Sepharose beads (GE Healthcare; 0.5 mL per liter of bacterial culture). The beads were washed four times with 50 mL of TNGMT buffer (supplemented with 0.05% Triton X-100) and incubated for 20 minutes with 2 bed volumes of TNTGsh buffer (50 mM Tris pH 7.4, 150 mM NaCl, 40 mM GSH, 1mM TCEP). The slurry was passed through a table-top column and the flow-through was collected after a wash with 1 bed volume of TNTGsh elution buffer. Tobacco Etch Virus protease (TEV) was added (1:100 mol:mol) and the eluate was incubated overnight at 4°C to remove the GST tag. The eluted and cleaved proteins were concentrated and passed through a Superdex 200 10/300 GL gel filtration column (GE Healthcare) connected in series with a 1 mL GSTrap FF column (GE Healthcare) equilibrated in HKG buffer (50 mM HEPES-KOH pH 7.6, 150 mM KCl, 10% glycerol). For IRE1^LD^-cys, the buffer was supplemented with 1 mM TCEP and 0.1 mM EDTA. Appropriate fractions were collected, concentrated, and flash frozen.

IRE1^LD^-cys (UK1915) and IRE1^CLD^-cys (UK1998) were labeled with a 3-fold molar excess of biotin-maleimide (Sigma) to make IRE1^LD^-biotin and IRE1^CLD^-biotin, respectively. IRE1^LD^-cys was labeled with a 1:100 (mol:mol) ratio of Oregon Green-iodoacetic acid (ThermoFisher) to make IRE1^LD^-OG. The reaction proceeded at room temperature in the dark for two hours and was quenched by the addition of 5 mM DTT. The reaction mixture was passed through a CentiPure P10 gravity-desalting column (Generon) equilibrated in HKG buffer and through a Superdex 200 10/300 GL gel filtration column equilibrated in HKG buffer. Appropriate fractions were collected, concentrated, and flash frozen.

The IRE1^LD^ R234C (UK2048) and IRE1^LD^ S112C (UK2076) used to make IRE1^LD^-donor and IRE1^LD^-acceptor, respectively, were encoded on a pET-derived vector (Novagen) as a His-Smt3 fusion protein and expressed as described above. Cells were harvested in HNKIGT buffer (25 mM HEPES-KOH pH 7.5, 400 mM NaCl, 100 mM KCl, 25 mM imidazole, 10% glycerol, 1 mM TCEP) and lysed and clarified as above. The lysates were cleared by centrifugation at 20,000 *g* for 60 minutes. The supernatant was removed, supplemented with 0.5% (v/v) Triton X-100, and incubated for 60 minutes at 4°C with Ni-NTA Agarose beads (ThermoFisher; 0.75 mL per liter of bacterial culture). The beads were washed four times with 50 mL of HNKIGT buffer (supplemented with 0.05% Triton X-100) and eluted with HNKIGT buffer supplemented with 250 mM imidazole. The eluate was concentrated, passed through a gravity desalting column equilibrated with HKG supplemented with 1 mM TCEP and 0.1 mM EDTA, concentrated and labeled overnight at room temperature with a 3-fold molar excess of TAMRA-maleimide (Sigma) or Oregon Green-iodoacetic acid (ThermoFisher) to create IRE1^LD^ ^S112C^-TAM and IRE1^LD^ ^R234C^-OG, respectively. The reaction mixtures were quenched, passed through a gravity desalting column, and passed through an S200 column as described above and the appropriate fractions were concentrated and flash frozen. IRE1^CLD^-S112C (UK2117) and IRE1^CLD^-R234C (UK2118) were expressed, purified, and labeled similarly.

The IRE1^LD^ Q105C (UK2045) used to make disulfide-linked dimeric IRE1^LD^-bio was expressed as an His6-Smt3 fusion protein in Origami B(DE3) cells (Novagen) and purified without reducing agent as described above. Dimeric IRE1^LD^-Q105C and standard IRE1^LD^ (UK2007) were labeled at a 1:10 (mol:mol) ratio with biotin-NHS ester (Sigma) for one hour at room temperature to create disulfide-linked dimeric IRE1^LD^-bio and wild-type IRE1^LD^-bio, respectively. Reactions were quenched by the addition of 5 mM Tris-HCl pH 8.

##### Hamster ERdj4

ERdj4 and variants were expressed as fusion proteins with an N-terminal His6-Smt3 (UK2012 for WT, UK2040 for QPD) or with both an N-terminal His6-Smt3 and C-terminal MBP (UK2108 for WT, UK2119 for QPD). Proteins were expressed in Origami B(DE3) cells. ERdj4 proteins expressed in BL21 (DE3) cells were not soluble, suggesting that ERdj4 may have a stabilizing disulfide between its two cysteines therefore, no reducing agent was used in purification of Erdj4 or its variants. Cells were grown and lysed as described above for His6-Smt3 tagged proteins. Media of cells expressing His6-Smt3-ERdj4-AviTag (UK2098) was supplemented with 0.2 mM Biotin (to allow the endogenous biotinylation enzymes of the bacteria to biotinylate the Erdj4 protein). The lysates were purified by Ni affinity chromatography as described above. His6-Smt3-ERdj4 was aliquotted immediately after elution from the Ni matrix. It was found to precipitate immediately upon cleavage of Smt3 by Ulp1. His6-Smt3-ERdj4-MBP was loaded onto an S200 10/300 GL column equilibrated in HKG buffer. Fractions containing His6-Smt3-ERdj4-MBP were collected, aliquotted, and flash frozen.

##### Human GRP170

N-terminally His6-tagged human GRP170 (UK1264) was expressed in BL21 (DE3) cells and induced, lysed, and bound to a Ni-NTA agarose beads as described above however, no detergent was present in any of the buffers and all buffers contained 5 mM ATP. The beads were sequentially washed with two bed volumes of HNIGβA buffer (50 mM HEPES-KOH pH 7.4, 300 mM NaCl, 5% glycerol, 10 mM imidazole, 5 mM β-mercaptoethanol, 5 mM ATP) supplemented with 0.5 M NaCl, 3 mM Mg^2+^-ATP, 0.25 M Tris pH 7.5, and 35 mM imidazole. The protein was then eluted in buffer HNIGβA supplemented with 240 mM imidazole. The eluted protein was loaded onto an Superdex S200 10/300 GL column equilibrated in HKMA buffer (50 mM HEPES-KOH pH 7.4, 150 mM KCl, 10 mM MgCl_2_, 0.5 mM ATP). GRP170-containing fractions were aliquotted and flash frozen.

##### BiP

BiP and BiP variants were purified as previously described (Petrova et al., 2008, Preissler et al., 2015a). Briefly, His6-BiP (WT and variants) was expressed and purified from BL21 C3013 *E. coli* cells as described above for His6-Smt3-IRE1. Cells were lysed in TNGMTr buffer (50 mM Tris pH 7.4, 500 mM NaCl, 10% glycerol, 1 mM MgCl_2_, 0.2% (v/v) Triton X-100, 20 mM imidazole) containing protease inhibitors and DnaseI as above. Prior to elution of BiP from the Ni-NTA agarose beads, the beads were washed with TNGMTr sequentially supplemented with 30 mM imidazole, 1% (v/v) Triton X-100, 1 M NaCl, 5 mM Mg2+-ATP and 0.5 M Tris–HCl pH 7.5. BiP was eluted in TNGMIz buffer (50 mM Tris–HCl pH 7.5, 500 mM NaCl, 1 mM MgCl2, 10% (v/v) glycerol, 250 mM imidazole) and dialyzed against HKM buffer (50 mM HEPES-KOH pH 7.4, 150 mM KCl, 10 mM MgCl2).

BiP NBD was purified as described for His6-Smt3-IRE1. After elution the protein was concentrated and passed through a CentiPure gravity-desalting column into 50 mM HEPES pH 7.4, 150 mM KCl, 10% glycerol and 0.1 mM EDTA.

AMPylation of purified BiP proteins was performed as previously described with minor modifications (Preissler et al., 2015b). Purified BiP was incubated for 6 hours at 30°C with 0.25 mg bacterially expressed FICD^E234G^ per 20 mg of BiP protein in presence of 3 mM ATP in buffer I [25 mM HEPES-KOH pH 7.4, 100 mM KCl, 10 mM MgCl_2_, 1 mM CaCl_2_, 0.1% (v/v) Triton X-100] followed by binding to Ni-NTA agarose beads for 1 hour at 25°C. The beads were washed with buffer I, and eluted in buffer I containing 350 mM imidazole for 45 minutes at 25°C. The eluate was desalted using a CentriPure column equilibrated in HKM buffer.

##### ERdj6 J domain

The J domain of ERdj6 (UK185) was purified as previously described (Petrova et al., 2008). Briefly, the J domain of ERdj6 was expressed as a GST fusion protein and purified as described above for GST fusion proteins. The protein was eluted in buffer H [50 mM HEPES-KOH pH 7.4, 100 mM KCl, 4 mM MgCl_2_, 1 mM CaCl_2_, 0.1% (v/v) Triton X-100, 1 mM DTT, 10% (v/v) glycerol, 40 mM reduced glutathione] and dialyzed overnight against HKM buffer.

##### GADD34-bio

GADD34-bio (UK1920) was purified as previously described (Crespillo-Casado et al., 2017). Briefly, GADD34-bio (PPP1R15A) was purified as above for GST tagged proteins with the modification that the TNGMT lysis buffer was supplemented with 1 mM MnCl_2_. Following the intial GST based purification and overnight incubation with TEV, cleaved GADD34 was bound to amylose beads (New England Biolabs) for 1–2 hr at 4°C. The amylose beads were washed with TNGMT and protein eluted with HEPES buffer (20 mM HEPES, 100 mM NaCl, 0.2 mM CaCl2, 0.2 mM ATP, 0.2 mM TCEP, 0.5 mM MnCl2, 100 μM PMSF, 20 mTIU/ ml aprotonin, 2 μM leupeptin, and 2 μg/ml pepstatin) and 10 mM maltose. The eluted GADD34 was then biotinylated using BirA (BirA UK1881 purified as described above for GST fusion proteins) in the presence of 2 mM MgCl_2_, 2 mM ATP, 0.01% Triton X-100, excess biotin (1:2 molar ratio to substrate protein) and BirA (1/20^th^ molar ratio of substrate protein). Following biotinylation GADD34-bio was passed through a CentiPure gravity-desalting column into HEPES buffer to remove excess of biotin that would interfere with the Bio-Layer Interferometry measurements.

Table S5 lists the concentration of the proteins used in each experiment.

#### Streptavidin pull-down assays

##### Assessing ERdj4 loading BiP onto IRE1

Schema shown in Figure 4B. 20 μL Dynabeads MyOne Streptavidin C1 (Thermo Fisher Scientific) per sample were used. Reactions were carried out in 150 mM KCl, 50 mM HEPES-KOH pH 7.4, 10 mM MgCl_2_, 1 mM CaCl_2_, 0.1% Triton X-100. Reactions contained 5 μM IRE1^LD^-bio, 8 μM ERdj4 or variants, 30 μM BiP or variants, and 2 mM ATP. Reactions proceeded for 20 minutes at 30°C before quenching with an excess of ice cold 1 mM ADP and clarification at 21,130 *g* for 5 minutes, followed by the addition of magnetic beads to supernatant. Binding was carried out for 15 minutes before washes and elution in first 5 mM ATP followed by SDS sample buffer. Pull-down experiments with the nucleotide binding domain of BiP were conducted similarly, but the ATP elution was skipped.

##### Assessing ERdj4’s effect on IRE1 dimerization

Schema shown in Figure 4E. Beads were first preloaded with IRE1^LD^-bio and then washed extensively. Beads were then incubated with the reaction mixtures containing 0.5 μM IRE1^LD^-TAM with 8 μM ERdj4 or variants, 30 μM BiP or variants, and 2 mM ATP. Where indicated, reactions also contained 1 μM GRP170. Reactions were quenched as described above. The beads were washed and the protein was eluted in SDS sample buffer.

##### Assessing the disruption of IRE1 dimers

Schema shown in Figure 5C. Beads were first pre-loaded with IRE1^LD^-bio and then washed extensively. Beads were incubated with 0.5 μM IRE1^LD^-TAM for one hour at 30°C, washed extensively, and then incubated with a solution of 30 μM BiP, 8 μM ERdj4, 1 μM GRP170, and 2 mM ATP at 30°C for 15 minutes. The reaction was quenched as described above and the beads were washed and eluted in SDS sample buffer.

#### Size-exclusion chromatography

Samples were run through a SEC-3 300A, 4.6x300 mm column (Agilent 5190-2513) on an Agilent infinity HPLC system in HKM buffer (150 mM KCl, 50 mM HEPES-KOH pH 7.4, 10 mM MgCl_2_). Reactions proceeded in 20 μL for 20 minutes at 30°C before clarification at 21,130 *g* for 5 minutes and subsequent injection.

#### Bio-Layer Interferometry experiments

Experiments were performed on an Octet RED96 (Pall ForteBio) in HKMGTr buffer (50 mM HEPES-KOH pH 7.6, 150 mM KCl, 10 mM MgCl_2_, 10% glycerol, 0.05% Triton X-100).

In the sequential dipping experiment (Figures 5A and S4C), streptavidin biosensors were loaded with IRE1^LD^-bio or GADD34-bio to approximately 1 nm shift, washed in buffer, and then sequentially dipped in wells containing 1.5 μM Smt3-ERdj4 or variants, 1 μM BiP with no nucleotide, 1μM BiP^T229A^ with 2 mM ATP, 1 μM BiP^V461F^ with 2 mM ATP, and 1 μM BiP (Figure 5A) or BiP at the indicated concentration (Figure S4C) with 2 mM ATP. Data were decimated, background-subtracted, and normalized to the signal after the first wash step.

In the titration experiment (Figure S4A), streptavidin biosensors were loaded with Smt3-ERdj4-bio to approximately 1 nm shift, washed in buffer, and then dipped in wells containing the indicated concentration of IRE1^LD^. Data were decimated and normalized to the signal after the first wash step.

In the sequential dipping experiment (Figure S4B), streptavidin biosensors were loaded with IRE1^LD^-bio to approximately 1 nm shift, washed in buffer, and then dipped in wells containing the indicated concentration of Smt3-ERdj4. Data were decimated, background subtracted, and normalized to the signal after the first wash step.

In the elution experiments (Figure 5B), streptavidin biosensors were loaded with IRE1^LD^-bio to a shift of approximately 7.5 nm. The biosensors were washed in buffer and then dipped in wells containing 1.2 μM Smt3-ERdj4. The biosensors were washed in buffer and then dipped in wells containing either: 6 μM BiP with no ATP, 6 μM BiP with 2 mM ATP, or 6 μM BiP^V461F^ with 2 mM ATP. The biosensors were then washed in buffer with 2 mM ATP and the was protein eluted in SDS sample buffer.

In the dimeric IRE1^LD^ experiment (Figure 5E), streptavidin biosensors were loaded with monomeric (UK2007) or disulphide-linked dimeric IRE1^LD^-bio (UK2045) to approximately 2 nm shift. The biosensors were washed in buffer and then incubated with 2.5 μM Smt3-ERdj4, and then incubated in buffer again.

In the sequential dipping and elution experiment (Figure S4D), streptavidin biosensors were loaded with IRE1^LD^-bio (UK2007) or IRE1^LD Q105C^-bio (UK2045) to a shift of approximately 3.5 nm. The biosensors were washed in buffer and then dipped in wells containing 1.7 μM Smt3-ERdj4. The biosensors were washed in buffer and then dipped in wells containing: 6 μM BiP with 2 mM ATP, or just buffer. The biosensors were then washed in buffer with 2 mM ATP and the protein eluted in SDS sample buffer and ran on a non-reducing SDS-PAGE gel. The BLI data was decimated and normalized to the signal after the first wash step.

#### FRET equilibrium experiments

In Figure 6A IRE1^LD^-donor and IRE1^LD^-acceptor were combined at a 1:2 ratio and incubated at room temperature in the dark for two hours. IRE1^LD^-donor and acceptor (0.2 μM total) was combined with unlabelled IRE1^LD^ at the specified concentration in HKMGTw buffer (50 mM HEPES-KOH pH 7.6, 150 mM KCl, 10 mM MgCl_2_, 10% glycerol, 0.05% TWEEN 20) and incubated for 3 hours. The samples were transferred to a black low volume 384-well plate and donor fluorescence was recorded with a CLARIOstar platereader (BMG), exciting at 470-15 nm and reading emission at 524-20 nm. Alternatively, IRE1^LD^-donor and acceptor was combined with BiP at the specified concentration in HKMGTw buffer and incubated for 24 hours. Fluorescence was recorded as described above. Fluorescence was normalized to that of IRE1^LD^-donor/IRE1^LD^ acceptor absent titrant.

#### FRET kinetic experiments

IRE1^LD^-donor and IRE1^LD^-acceptor were combined at a 1:2 ratio and incubated at room temperature in the dark for two hours. In Figure 6B BiP, Smt3-ERdj4-MBP, and IRE1^LD^-donor and acceptor were combined in HKMGTw buffer. The concentrations used were 30 μM BiP, 2.5 μM Smt3-ERdj4-MBP, and 0.2 μM IRE1^LD^-donor and acceptor. After incubation for 30 minutes, 2 mM ATP with an ATP regeneration system (8 mM phosphocreatine, 0.016 mg/ml creatine kinase) was added to initiate the reaction. In the indicated wells, 1 μM GRP170 was added along with ATP and the regeneration system. In Figure 6C, 50 μM BiP, 2.5 μM Smt3-ERdj4-MBP, and 0.2 μM equilibrated IRE1^LD^-donor and acceptor were combined in HKMGTw buffer. After incubation for 30 minutes, 2 mM ATP with the ATP regeneration system was added to initiate the reaction. At the indicated time, buffer, CH1 heptapeptide (HTFPAVL, a model BiP substrate) at the indicated concentration, and/or 2.5 μM ERdj6 J domain was added. In all kinetic experiments, donor fluorescence was followed with a CLARIOstar plate reader (excitation: 470-15 nm / emission: 524-20 nm) recording fluorescence every 30 s. Fluorescence was normalized to the level at t = 0.

### Quantification and Statistical Analysis

The GraphPad-Prism V7 software was used for all statistical analysis. Statistical details of experiments can be found in the figure legends.

Supplemental table S6 tabulates the number of times the key observation reported on in each of the paper’s 38 representative data panels has been reproduced

Figure 1A. XBP1s::Turquoise and CHOP::GFP reporter activity in CHO cells deleted in the indicated ER-localized J protein (ERdj). Bars show the median fluorescence (±SEM) from 20,000 cells, normalized to wild-type (WT).

Figure 1B. XBP1s::Turquoise and CHOP::GFP activity in CHO cells untreated or treated with the IRE1 inhibitor 4μ8C, which blocks IRE1-dependent CHOP activation. Fluorescence normalized to WT. (Bars show the mean (±SD) of median fluorescence obtained from independent experiments (n = 3) where each median was determined from 20,000 cells, ^∗∗∗^p = 0.0005, repeated-measurements one-way ANOVA, Dunnett’s multiple corrections test).

Figure 1F. Bars show mean ratio of BiP to IRE1 signal (±SD) from 6 independent experiments (n = 6) as in Figure 1E, ^∗^p = 0.0118 by parametric ratio paired Student’s t test.

Figure S1A. Plot of tunicamycin (Tm) concentration-dependent changes in XBP1s::Turquoise and CHOP::GFP reporter gene activity in wild-type CHO cells. Shown is the median fluorescence value (normalized to the untreated sample) obtained from 10,000 cells in experimental triplicates and the fit to sigmoidal dose-response curve.

Figure 2C. Bars show mean ratio of BiP to IRE1^LD^-GST signal (±SD) from 4 independent experiments (n = 4), as in Figure 2B, ^∗∗^p = 0.0048 by parametric ratio paired Student’s t test.

Figure 3F. Bars show mean ratio of disulfide-bound IRE1^LD^ Q105C-GST dimer to free thiol (±SD) from 6 independent experiments (n = 6) as shown in Figure 2D, ^∗∗∗∗^p < 0.0001 by unpaired Student’s t test with Welch’s correction.

Figure 4H. Bars show mean BiP signal recovered with IRE1^LD^-bio (±SD) from 3 independent experiments (n = 3) as shown in Figure 4G, ^∗^p = 0.0223 by Student’s paired ratio t test.

Figure S3C. Bars show mean IRE1^LD^-TAM signal recovered with IRE1^LD^-bio (±SD) from 3 independent experiments (n = 3) as shown in Figure 4G, ^∗^p = 0.0293 by Student’s paired ratio t test.

Figure 5D. Bars show mean IRE1^LD^-TAM signal recovered with IRE1^LD^-bio (±SD) from 4 independent experiments (n = 4), ^∗∗∗^p = 0.001 by parametric student’s paired ratio t test.

Figure 6A. IRE1^LD^-OG^488^ donor fluorescence (blue trace), mean values ± SD from 3 independent experiments

## Author Contributions

N.A.-W. conceived, initiated, and led the project, designed and conducted most of the cell-based and protein co-purification experiments, thereby discovering the role of ERdj4 in catalyzing the monomerization of IRE1, created reagents for *in vitro* experiments, analyzed and interpreted the data, prepared the figures, and wrote the manuscript. R.A.S. conceived, designed, and implemented the biophysical assays for measuring IRE1 oligomerization and the IRE1-ERdj4 interaction, created reagents for all *in vitro* experiments, analyzed and interpreted the experiments, and wrote the manuscript. M.J.K. systematically inactivated the eight known ERdj proteins and evaluated the consequences in cells. C.R. contributed to execution and interpretation of experiments with the ERdj protein mutants, supervising M.J.K. in this task. S.P. designed the systematic ERdj inactivation strategy and generated BiP mutants. H.P.H. measured the formation of the ER stress-induced disulfide in endogenous IRE1^Q105C^. D.R. conceived and oversaw the project, constructed plasmid DNA, interpreted the data, and wrote the manuscript.

## References

[bib1] Abravaya K., Myers M.P., Murphy S.P., Morimoto R.I. (1992). The human heat shock protein hsp70 interacts with HSF, the transcription factor that regulates heat shock gene expression. Genes Dev..

[bib2] Adamson B., Norman T.M., Jost M., Cho M.Y., Nuñez J.K., Chen Y., Villalta J.E., Gilbert L.A., Horlbeck M.A., Hein M.Y. (2016). A Multiplexed Single-Cell CRISPR Screening Platform Enables Systematic Dissection of the Unfolded Protein Response. Cell.

[bib3] Avezov E., Cross B.C., Kaminski Schierle G.S., Winters M., Harding H.P., Melo E.P., Kaminski C.F., Ron D. (2013). Lifetime imaging of a fluorescent protein sensor reveals surprising stability of ER thiol redox. J. Cell Biol..

[bib4] Behnke J., Feige M.J., Hendershot L.M. (2015). BiP and its nucleotide exchange factors Grp170 and Sil1: mechanisms of action and biological functions. J. Mol. Biol..

[bib5] Bertolotti A., Zhang Y., Hendershot L.M., Harding H.P., Ron D. (2000). Dynamic interaction of BiP and ER stress transducers in the unfolded-protein response. Nat. Cell Biol..

[bib6] Calfon M., Zeng H., Urano F., Till J.H., Hubbard S.R., Harding H.P., Clark S.G., Ron D. (2002). IRE1 couples endoplasmic reticulum load to secretory capacity by processing the *XBP-1* mRNA. Nature.

[bib7] Carrara M., Prischi F., Nowak P.R., Ali M.M. (2015). Crystal structures reveal transient PERK luminal domain tetramerization in endoplasmic reticulum stress signaling. EMBO J..

[bib8] Carrara M., Prischi F., Nowak P.R., Kopp M.C., Ali M.M. (2015). Noncanonical binding of BiP ATPase domain to Ire1 and Perk is dissociated by unfolded protein CH1 to initiate ER stress signaling. eLife.

[bib9] Cox J.S., Walter P. (1996). A novel mechanism for regulating activity of a transcription factor that controls the unfolded protein response. Cell.

[bib10] Cox J.S., Shamu C.E., Walter P. (1993). Transcriptional induction of genes encoding endoplasmic reticulum resident proteins requires a transmembrane protein kinase. Cell.

[bib11] Cox J.S., Chapman R.E., Walter P. (1997). The unfolded protein response coordinates the production of endoplasmic reticulum protein and endoplasmic reticulum membrane. Mol. Biol. Cell.

[bib12] Credle J.J., Finer-Moore J.S., Papa F.R., Stroud R.M., Walter P. (2005). On the mechanism of sensing unfolded protein in the endoplasmic reticulum. Proc. Natl. Acad. Sci. USA.

[bib13] Crespillo-Casado A., Chambers J.E., Fischer P.M., Marciniak S.J., Ron D. (2017). PPP1R15A-mediated dephosphorylation of eIF2α is unaffected by Sephin1 or Guanabenz. eLife.

[bib14] Cross B.C., Bond P.J., Sadowski P.G., Jha B.K., Zak J., Goodman J.M., Silverman R.H., Neubert T.A., Baxendale I.R., Ron D., Harding H.P. (2012). The molecular basis for selective inhibition of unconventional mRNA splicing by an IRE1-binding small molecule. Proc. Natl. Acad. Sci. USA.

[bib15] De Los Rios P., Barducci A. (2014). Hsp70 chaperones are non-equilibrium machines that achieve ultra-affinity by energy consumption. eLife.

[bib16] Dong M., Bridges J.P., Apsley K., Xu Y., Weaver T.E. (2008). ERdj4 and ERdj5 are required for endoplasmic reticulum-associated protein degradation of misfolded surfactant protein C. Mol. Biol. Cell.

[bib17] Freiden P.J., Gaut J.R., Hendershot L.M. (1992). Interconversion of three differentially modified and assembled forms of BiP. EMBO J..

[bib18] Fritz J.M., Weaver T.E. (2014). The BiP cochaperone ERdj4 is required for B cell development and function. PLoS ONE.

[bib19] Gardner B.M., Walter P. (2011). Unfolded proteins are Ire1-activating ligands that directly induce the unfolded protein response. Science.

[bib20] Ghaemmaghami S., Huh W.K., Bower K., Howson R.W., Belle A., Dephoure N., O’Shea E.K., Weissman J.S. (2003). Global analysis of protein expression in yeast. Nature.

[bib21] Harding H.P., Zhang Y., Ron D. (1999). Protein translation and folding are coupled by an endoplasmic-reticulum-resident kinase. Nature.

[bib22] Kampinga H.H., Craig E.A. (2010). The HSP70 chaperone machinery: J proteins as drivers of functional specificity. Nat. Rev. Mol. Cell Biol..

[bib23] Karagöz G.E., Acosta-Alvear D., Nguyen H.T., Lee C.P., Chu F., Walter P. (2017). An unfolded protein-induced conformational switch activates mammalian IRE1. eLife.

[bib24] Kim M.S., Pinto S.M., Getnet D., Nirujogi R.S., Manda S.S., Chaerkady R., Madugundu A.K., Kelkar D.S., Isserlin R., Jain S. (2014). A draft map of the human proteome. Nature.

[bib25] Kimata Y., Kimata Y.I., Shimizu Y., Abe H., Farcasanu I.C., Takeuchi M., Rose M.D., Kohno K. (2003). Genetic evidence for a role of BiP/Kar2 that regulates Ire1 in response to accumulation of unfolded proteins. Mol. Biol. Cell.

[bib26] Kinoshita E., Kinoshita-Kikuta E., Koike T. (2009). Separation and detection of large phosphoproteins using Phos-tag SDS-PAGE. Nat. Protoc..

[bib27] Kozutsumi Y., Segal M., Normington K., Gething M.J., Sambrook J. (1988). The presence of malfolded proteins in the endoplasmic reticulum signals the induction of glucose-regulated proteins. Nature.

[bib28] Le Gall S., Neuhof A., Rapoport T. (2004). The endoplasmic reticulum membrane is permeable to small molecules. Mol. Biol. Cell.

[bib29] Lee A.H., Iwakoshi N.N., Glimcher L.H. (2003). XBP-1 regulates a subset of endoplasmic reticulum resident chaperone genes in the unfolded protein response. Mol. Cell. Biol..

[bib30] Lee K.P.K., Dey M., Neculai D., Cao C., Dever T.E., Sicheri F. (2008). Structure of the dual enzyme Ire1 reveals the basis for catalysis and regulation in nonconventional RNA splicing. Cell.

[bib31] Liu C.Y., Schröder M., Kaufman R.J. (2000). Ligand-independent dimerization activates the stress response kinases IRE1 and PERK in the lumen of the endoplasmic reticulum. J. Biol. Chem..

[bib32] Ma K., Vattem K.M., Wek R.C. (2002). Dimerization and release of molecular chaperone inhibition facilitate activation of eukaryotic initiation factor-2 kinase in response to endoplasmic reticulum stress. J. Biol. Chem..

[bib33] Marcinowski M., Höller M., Feige M.J., Baerend D., Lamb D.C., Buchner J. (2011). Substrate discrimination of the chaperone BiP by autonomous and cochaperone-regulated conformational transitions. Nat. Struct. Mol. Biol..

[bib34] Mayer M.P. (2013). Hsp70 chaperone dynamics and molecular mechanism. Trends Biochem. Sci..

[bib35] Misselwitz B., Staeck O., Rapoport T.A. (1998). J proteins catalytically activate Hsp70 molecules to trap a wide range of peptide sequences. Mol. Cell.

[bib36] Mori K., Ma W., Gething M.J., Sambrook J. (1993). A transmembrane protein with a cdc2+/CDC28-related kinase activity is required for signaling from the ER to the nucleus. Cell.

[bib37] Ng D.T., Watowich S.S., Lamb R.A. (1992). Analysis in vivo of GRP78-BiP/substrate interactions and their role in induction of the GRP78-BiP gene. Mol. Biol. Cell.

[bib38] Oikawa D., Kimata Y., Kohno K., Iwawaki T. (2009). Activation of mammalian IRE1alpha upon ER stress depends on dissociation of BiP rather than on direct interaction with unfolded proteins. Exp. Cell Res..

[bib39] Okamura K., Kimata Y., Higashio H., Tsuru A., Kohno K. (2000). Dissociation of Kar2p/BiP from an ER sensory molecule, Ire1p, triggers the unfolded protein response in yeast. Biochem. Biophys. Res. Commun..

[bib40] Petrova K., Oyadomari S., Hendershot L.M., Ron D. (2008). Regulated association of misfolded endoplasmic reticulum lumenal proteins with P58/DNAJc3. EMBO J..

[bib41] Pincus D., Chevalier M.W., Aragón T., van Anken E., Vidal S.E., El-Samad H., Walter P. (2010). BiP binding to the ER-stress sensor Ire1 tunes the homeostatic behavior of the unfolded protein response. PLoS Biol..

[bib42] Plumb R., Zhang Z.R., Appathurai S., Mariappan M. (2015). A functional link between the co-translational protein translocation pathway and the UPR. eLife.

[bib43] Preissler S., Chambers J.E., Crespillo-Casado A., Avezov E., Miranda E., Perez J., Hendershot L.M., Harding H.P., Ron D. (2015). Physiological modulation of BiP activity by *trans*-protomer engagement of the interdomain linker. eLife.

[bib44] Preissler S., Rato C., Chen R., Antrobus R., Ding S., Fearnley I.M., Ron D. (2015). AMPylation matches BiP activity to client protein load in the endoplasmic reticulum. eLife.

[bib45] Ran F.A., Hsu P.D., Wright J., Agarwala V., Scott D.A., Zhang F. (2013). Genome engineering using the CRISPR-Cas9 system. Nat. Protoc..

[bib46] Rodriguez F., Arsène-Ploetze F., Rist W., Rüdiger S., Schneider-Mergener J., Mayer M.P., Bukau B. (2008). Molecular basis for regulation of the heat shock transcription factor sigma32 by the DnaK and DnaJ chaperones. Mol. Cell.

[bib47] Ron D., Habener J.F. (1992). CHOP, a novel developmentally regulated nuclear protein that dimerizes with transcription factors C/EBP and LAP and functions as a dominant-negative inhibitor of gene transcription. Genes Dev..

[bib48] Ronda C., Pedersen L.E., Hansen H.G., Kallehauge T.B., Betenbaugh M.J., Nielsen A.T., Kildegaard H.F. (2014). Accelerating genome editing in CHO cells using CRISPR Cas9 and CRISPy, a web-based target finding tool. Biotechnol. Bioeng..

[bib49] Sekine Y., Zyryanova A., Crespillo-Casado A., Amin-Wetzel N., Harding H.P., Ron D. (2016). Paradoxical Sensitivity to an Integrated Stress Response Blocking Mutation in Vanishing White Matter Cells. PLoS ONE.

[bib50] Shamu C.E., Walter P. (1996). Oligomerization and phosphorylation of the Ire1p kinase during intracellular signaling from the endoplasmic reticulum to the nucleus. EMBO J..

[bib51] Shen Y., Meunier L., Hendershot L.M. (2002). Identification and characterization of a novel endoplasmic reticulum (ER) DnaJ homologue, which stimulates ATPase activity of BiP in vitro and is induced by ER stress. J. Biol. Chem..

[bib52] Sundaram A., Plumb R., Appathurai S., Mariappan M. (2017). The Sec61 translocon limits IRE1α signaling during the unfolded protein response. eLife.

[bib53] Tomoyasu T., Ogura T., Tatsuta T., Bukau B. (1998). Levels of DnaK and DnaJ provide tight control of heat shock gene expression and protein repair in Escherichia coli. Mol. Microbiol..

[bib54] Wall D., Zylicz M., Georgopoulos C. (1994). The NH2-terminal 108 amino acids of the Escherichia coli DnaJ protein stimulate the ATPase activity of DnaK and are sufficient for lambda replication. J. Biol. Chem..

[bib55] Walter P., Ron D. (2011). The unfolded protein response: from stress pathway to homeostatic regulation. Science.

[bib56] Wang M., Kaufman R.J. (2016). Protein misfolding in the endoplasmic reticulum as a conduit to human disease. Nature.

[bib57] Wang X.Z., Harding H.P., Zhang Y., Jolicoeur E.M., Kuroda M., Ron D. (1998). Cloning of mammalian Ire1 reveals diversity in the ER stress responses. EMBO J..

[bib58] Xing Y., Böcking T., Wolf M., Grigorieff N., Kirchhausen T., Harrison S.C. (2010). Structure of clathrin coat with bound Hsc70 and auxilin: mechanism of Hsc70-facilitated disassembly. EMBO J..

[bib59] Yang L., Xue Z., He Y., Sun S., Chen H., Qi L. (2010). A Phos-tag-based approach reveals the extent of physiological endoplasmic reticulum stress. PLoS ONE.

[bib60] Yoshida H., Matsui T., Yamamoto A., Okada T., Mori K. (2001). XBP1 mRNA is induced by ATF6 and spliced by IRE1 in response to ER stress to produce a highly active transcription factor. Cell.

[bib61] Zhou J., Liu C.Y., Back S.H., Clark R.L., Peisach D., Xu Z., Kaufman R.J. (2006). The crystal structure of human IRE1 luminal domain reveals a conserved dimerization interface required for activation of the unfolded protein response. Proc. Natl. Acad. Sci. USA.

