# Supplementary material for: A J-Protein Co-chaperone Recruits BiP to Monomerize IRE1 and Repress the Unfolded Protein Response
Source: Cell. 2017 Dec 14;171(7):1625–1637.e13. doi: 10.1016/j.cell.2017.10.040 (PMC5733394; doi:10.1016/j.cell.2017.10.040)
Supplement: Document S1. Tables S1–S6 [file mmc1.pdf]

Cell, Volume *171*

Supplemental Information

A J-Protein Co-chaperone Recruits BiP to Monomerize IRE1 and Repress the Unfolded Protein Response

Niko Amin-Wetzel, Reuben A. Saunders, Maarten J. Kamphuis, Claudia Rato, Steffen Preissler, Heather P. Harding, and David Ron

Table S1: Plasmids used in this study, related to STAR methods and Key resources table

ID	Plasmid name	Description	Comments/reference	Figure
UK173	haBiP_27-654_pQE10	N-terminally His6-tagged hamster BiP	PMID: 18923430	4A,C,D,F,G;S3A,B;5A,B,D,E,F;S4B,C,D;6A,B,C;S5
UK182	haBiP_27-654_V461F_pQE10	N-terminally His6-tagged hamster BiP V461F	PMID: 18923430	4C,F;5A,B;S4C;6B
UK838	haBiP_27-654_T229A_pQE10	N-terminally His6-tagged hamster BiP T229A	PMID: 26673894	4C,F;5A;S4C;6B
UK1047	PERK_GST_pCDNA3	mPERK 1-585 (LD + TM) fused to GST	This paper. Thanks to Yuhong Zhang.	2D
UK1070	pFLAGM1_mP58dSP1_CMV1	FLAGM1 mP58 27_504 (ΔSP) in pFLAG_pCMV1	This paper. Thanks to Yusuke Sekine.	2E
UK1314	pCEFL_mCherry_3XFLAG_C	pCEFL with 3XFLAG_C tagged from mCherry-tagged plasmid	PMID: 25858979	1C;S1B
UK1558	IRE1_Q105C_g1b_pSpCas9(BB)-2A-Puro	Cas9 targetted to CHO IRE1 exon 5 to introduce indel	This paper	Used to generate ΔIRE1 and IRE1-Q105C cell lines for 1D,E;2H;3B,C;S2
UK1559	IRE1_Q105C_g2a_pSpCas9(BB)-2A-Puro	Cas9 targetted to CHO IRE1 exon 5 to introduce Q105C mutation via HDR	This paper	Used to generate IRE1-Q105C cell line for 3B,C;S2
UK1610	pSpCas9(BB)-2A-mCherry_V2	Modified pSpCas9(BB)-2A vector to express mCherry together with guide RNA/Cas9	This paper	Used to generate cell line for 1A
UK1682	ERdj1_g1_pSpCas9(BB)-2A-mCherry	mCherry-tagged CRISPR plasmid (UK1610) for targeting hamster ERdj1 (primers 1274 and 1275)	ERdj1 (Mtj1) DNAJC1 Gene ID: 100751540 This paper	Used to generate cell line for 1A
UK1683	ERdj1_g2_pSpCas9(BB)-2A-mCherry	mCherry-tagged CRISPR plasmid (UK1610) for targeting hamster ERdj1 (primers 1276 and 1277)		Used to generate cell line for 1A
UK1684	ERdj2_g1_pSpCas9(BB)-2A-mCherry	mCherry-tagged CRISPR plasmid (UK1610) for targeting hamster ERdj2 (primers 1278 and 1279)	ERdj2 (Sec63) Gene ID: 100775020 This paper	Used to generate cell line for 1A,B
UK1685	ERdj3_g1_pSpCas9(BB)-2A-mCherry	mCherry-tagged CRISPR plasmid (UK1610) for targeting hamster ERdj3 (primers 1280 and 1281)	ERdj3 (HEDJ) Dnajb11 Gene ID: 100765455 This paper	Used to generate cell line for 1A
UK1686	ERdj3_g2_pSpCas9(BB)-2A-mCherry	mCherry-tagged CRISPR plasmid (UK1610) for targeting hamster ERdj3 (primers 1282 and 1283)		Used to generate cell line for 1A
UK1687	ERdj4_g1_pSpCas9(BB)-2A-mCherry	mCherry-tagged CRISPR plasmid (UK1610) for targeting hamster ERdj4 (primers 1284 and 1285)	ERdj4 (MDG-1) Dnajb9 Gene ID: 100763648 This paper	Used to generate cell line for 1A,B,C,D,E;S1B,C,D;2B,D,E,F,G;3E
UK1688	ERdj4_g2_pSpCas9(BB)-2A-mCherry	mCherry-tagged CRISPR plasmid (UK1610) for targeting hamster ERdj4 (primers 1286 and 1287)		Used to generate cell line for 1A,B,C,D,E;S1B,C,D;2B,D,E,F,G;3E
UK1689	ERdj5_g1_pSpCas9(BB)-2A-mCherry	mCherry-tagged CRISPR plasmid (UK1610) for targeting hamster ERdj5 (primers 1288 and 1289)	ERdj5 (JPDI) Dnajc10 Gene ID: 100689230 This paper	Used to generate cell line for 1A
UK1690	ERdj5_g2_pSpCas9(BB)-2A-mCherry	mCherry-tagged CRISPR plasmid (UK1610) for targeting hamster ERdj5 (primers 1290 and 1291)		Used to generate cell line for 1A
UK1691	ERdj6_g1_pSpCas9(BB)-2A-mCherry	mCherry-tagged CRISPR plasmid (UK1610) for targeting hamster ERdj6 (primers 1292 and 1293)	ERdj6 (p58IPK) Dnajc3 Gene ID: 100766172 This paper	Used to generate cell line for 1A
UK1692	ERdj6_g2_pSpCas9(BB)-2A-mCherry	mCherry-tagged CRISPR plasmid (UK1610) for targeting hamster ERdj6 (primers 1294 and 1295)		Used to generate cell line for 1A
UK1693	ERdj7_g1_pSpCas9(BB)-2A-mCherry	mCherry-tagged CRISPR plasmid (UK1610) for targeting hamster ERdj7 (primers 1296 and 1297)	ERdj7 Dnajc25 Gene ID: 100756280 This paper	Used to generate cell line for 1A
UK1694	ERdj7_g2_pSpCas9(BB)-2A-mCherry	mCherry-tagged CRISPR plasmid (UK1610) for targeting hamster ERdj7 (primers 1298 and 1299)		Used to generate cell line for 1A
UK1703	hIRE1α_19-486_dC_GST_del3UTR _pCDNA3	GST tagged hIRE1α lacking lumenal domain cysteines for expression in mammalian cells	This paper	2B,D,E,F,G
UK1765	hIRE1α_19-486_dC_dRVI_II_GST_del3UTR _pCDNA3	GST tagged hIRE1α with a deletion (403-441) lacking lumenal domain cysteines for expression in mammalian cells	This paper	2F
UK1739	pCEFL_mCherry_CHO_ERdj4_3XFLAG_C	Mammalian expression of hamster 3xFLAG-tagged ERdj4 from mCherry-tagged plasmid	This paper	1C;S1B;2B,C,D,E,F,G
UK1754	pCEFL_mCherry_CHO_QDL_ERdj4_3XFLAG_C	Mammalian expression of hamster QPD mutant3xFLAG-tagged ERdj4 from mCherry-tagged plasmid	This paper	1C;S1B;2B
UK1861	ERdj8_guide1_pSpCas(BB)-2A-mCherry_V2	mCherry-tagged CRISPR plasmid (UK1610) for targeting hamster ERdj8 (oligos 1618 and 1619)	ERdj8 Dnajc16 Gene ID: 100751780 This paper	Used to generate cell line for 1A
UK1862	hIRE1α_19-486_dC_Q105C_GST_del3UTR _pCDNA3	GST tagged hIRE1α lacking lumenal domain cysteines containing Q105C for expression in mammalian cells	This paper	3E
UK1915	pGEX_GST_TEV_hIRE1a_LDΔC_24-442_D443C	N-terminally GST-tagged cysteine free human Ire1LD 24-442 with a C-terminal cysteine	This paper	4A,C,D,F,G;S3A;5A,B,C,F;S4B,C
UK1920	huPPP1R15A_533_624_malE_pGEX_TEV_AviTag	N-tern AviTagged human GADD34 533–624	This paper	5A
UK2007	pGEX_GST_TEV_hIRE1a_LDΔC_24-443	N-terminally GST-tagged cysteine free human Ire1LD 24-442	This paper	5E;S4D
UK2008	haBiP_27-405_NBD_pQE10	Bacterial expression of BiP NBD	This paper	S3A
UK2012	Smt3_cgERdJ4_24-222_pET-21a	N-terminally His6-Smt3-tagged chinese hamster ERdj4 24-222	This paper	4A,C,D,F,G;S3A;5A,B,D,E,F;S4B,C,D
UK2040	Smt3_QPD_cgERdJ4_24-222_pET-21a	N-terminally His6-Smt3-tagged chinese hamster ERdj4 24-222, H54Q	This paper	4C,F;5A
UK2041	Smt3_J4_domain_24-90_pET-21a	N-terminally His6-Smt3-tagged chinese hamster ERdj4 24-90	This paper	4C,F;5A;6B
UK2045	pET22b_H7_Smt3_Ire1a_LDΔC_24_443 Q105C	N-terminally His6-Smt3-tagged cysteine free human Ire1LD 24-443, Q105C	This paper	5E;S4D
UK2047	pET22b_H7_Smt3_Ire1a_LDΔC_24_443 S152C	Bacterial expression of Smt3-tagged cysteine-free Ire1a LDΔC S152C	This paper	S3B
UK2048	pET22b_H7_Smt3_Ire1a_LDΔC_24_443 R234C	N-terminally His6-Smt3-tagged cysteine free human Ire1LD 24-443, R234C	This paper	6A,B,C
UK2076	pET22b_H7_Smt3_Ire1a_LDΔC_24_443 S112C	N-terminally His6-Smt3-tagged cysteine free human Ire1LD 24-443, S112C	This paper	6A,B,C
UK2098	Smt3_cgERdJ4_24-222_AviTag_pET21a	N-terminally His6-Smt3-tagged and C-terminally avi-tagged chinese hamster ERdj4 24-222, H54Q	This paper	S4A
UK2108	Smt3_cgERdJ4_24-222_GS6_MalE_pET21a	N-terminally His6-Smt3-tagged and C-terminally MBP-tagged chinese hamster ERdj4 24-222	This paper	S3B;6B,C;S5
UK2117	pET22b_H7_Smt3_Ire1a_LDΔC_24_390 R234C	N-terminally His6-Smt3-tagged cysteine free human Ire1CLD 24-390, R234C	This paper	S5
UK2118	pET22b_H7_Smt3_Ire1a_LDΔC_24_390 S112C	N-terminally His6-Smt3-tagged cysteine free human Ire1CLD 24-390, S112C	This paper	S5
UK2119	Smt3_cgERdJ4_24-222_QPD_GS6_MalE_pET21a	N-terminally His6-Smt3-tagged and C-terminally MBP-tagged chinese hamster ERdj4 24-222, H54Q	This paper	6B
UK2132	pCMV1_SP_QPD_J4_mCherry_KDEL	mammalian expression CHO QPD J4 mCherry with KDEL	This paper	S1C
UK2080	pCMV1_SP_J4_mCherry_KDEL	mammalian expression CHO ERdj4 J domain alone mCherry with KDEL	This paper	S1C
UK2127	pCMV1_SP_ERdJ4_3xFLAG_mCherry_KDEL	mammalian expression CHO ERdj4 3xFLAG mCherry with KDEL	This paper	S1C
UK888	pFLAG_mCherry_KDEL_CMV1	mammalian expression of SP-FLAGM1-mCherry-KDEL (ER localized)	This paper	S1C
UK1881	EcBirA_WT_pGEX_TEV	Bacterial expression of fastidious E. coli BirA biotin ligase (R118 intact)		Used for in vitro biotinylation of UK1920

Table S2: Oligonucleotides used in this study, related to STAR methods and Key resources table

ID	Primer name	Sequence	Description
1097	IRE1_Q105C_knit_2as	ATGGGCTAGCACAGACTAATTCTGGGATGGTAAAGGGAAGTTTC	Primer for Q105C repair template of Q105 region of CHO IRE1
1098	IRE1_Q105C_knit_3s	CAGAATTAGTCTGTGCTAGCCCATCCCGAAGTTCAGATGGAATCCTCTAC	Primer for Q105C repair template of Q105 region of CHO IRE1
1100	IRE1_Q105_region_1s	AGCCTCCATCTGCAGTGTGCTTCTCTG	Oligos for genotyping of CHO IRE1 Q105 region
1101	IRE1_Q105_region_2as	CACAACTTTCCCAGATTCCAGGATTCACTGTC	Oligos for genotyping of CHO IRE1 Q105 region
1116	IRE1_Q105_region_3s	GCTAGAAATAGTGTGGAGTGATCAG	Oligos for generating repair template of CHO IRE1 Q105 region
1117	IRE1_Q105_region_4as	AACTATTCCCAGGTCACAGGTTATA	Oligos for generating repair template of CHO IRE1 Q105 region
1125	IRE1_Q105_region_1s [5'-6FAM]	AGCCTCCATCTGCAGTGTGCTTCTCTG	Oligo 1100 with 5'FAM for genotyping of CHO IRE1 Q105 region
1274	ERdj1_guide1_S	CACCGGGCCCCGGGCGCCGGCGCTG	CRISPR-Cas9 guide oligonucleotide ERdj1
1275	ERdj1_guide1_AS	AAACCAGCGCCGGCGCCCGGGGCCC	CRISPR-Cas9 guide oligonucleotide ERdj1
1276	ERdj1_guide2_S	CACCGGGCCGCTGTCGTCCTCCGTT	CRISPR-Cas9 guide oligonucleotide ERdj1
1277	ERdj1_guide2_AS	AAACAACGGAGGACGACAGCGGCCC	CRISPR-Cas9 guide oligonucleotide ERdj1
1278	ERdj2_guide1_S	CACCGTCCAAGTTTAATACTTCATA	CRISPR-Cas9 guide oligonucleotide ERdj2
1279	ERdj2_guide1_AS	AAACTATGAAGTATTAAACTTGGAC	CRISPR-Cas9 guide oligonucleotide ERdj2
1280	ERdj3_guide1_S	CACCGGAAATCTCGCCTGAGAGGAC	CRISPR-Cas9 guide oligonucleotide ERdj3
1281	ERdj3_guide1_AS	AAACGTCCTCTCAGGCGAGATTTCC	CRISPR-Cas9 guide oligonucleotide ERdj3
1282	ERdj3_guide2_S	CACCGGCCTCGGAGTGCCTCCGTAA	CRISPR-Cas9 guide oligonucleotide ERdj3
1283	ERdj3_guide2_AS	AAACTTACGGAGGCACTCCGAGGCC	CRISPR-Cas9 guide oligonucleotide ERdj3
1284	ERdj4_guide1_S	CACCGCTCAGAGCGACAAATCAAGA	CRISPR-Cas9 guide oligonucleotide ERdj4
1285	ERdj4_guide1_AS	AAACTCTTGATTTGTCGCTCTGAGC	CRISPR-Cas9 guide oligonucleotide ERdj4
1286	ERdj4_guide2_S	CACCGGATATCATAGTAGCTCTTTG	CRISPR-Cas9 guide oligonucleotide ERdj4
1287	ERdj4_guide2_AS	AAACCAAAGAGCTACTATGATATCC	CRISPR-Cas9 guide oligonucleotide ERdj4
1288	ERdj5_guide1_S	CACCGCTTCAGTGCTAATTTCTTAA	CRISPR-Cas9 guide oligonucleotide ERdj5
1289	ERdj5_guide1_AS	AAACTTAAGAAATTAGCACTGAAGC	CRISPR-Cas9 guide oligonucleotide ERdj5
1290	ERdj5_guide2_S	CACCGTTCTCTACTACTTGCAGTTT	CRISPR-Cas9 guide oligonucleotide ERdj5
1291	ERdj5_guide2_AS	AAACAAACTGCAAGTAGTAGAGAAC	CRISPR-Cas9 guide oligonucleotide ERdj5
1292	ERdj6_guide1_S	CACCGGTGACTGTTTCAGTAACCGC	CRISPR-Cas9 guide oligonucleotide ERdj6
1293	ERdj6_guide1_AS	AAACGCGGTTACTGAAACAGTCACC	CRISPR-Cas9 guide oligonucleotide ERdj6
1294	ERdj6_guide2_S	CACCGCTAAACCTTCCCGAATCTGC	CRISPR-Cas9 guide oligonucleotide ERdj6
1295	ERdj6_guide2_AS	AAACGCAGATTCGGGAAGGTTTAGC	CRISPR-Cas9 guide oligonucleotide ERdj6
1296	ERdj7_guide1_S	CACCGGAAAGATTATGACTACATGC	CRISPR-Cas9 guide oligonucleotide ERdj7
1297	ERdj7_guide1_AS	AAACGCATGTAGTCATAATCTTTCC	CRISPR-Cas9 guide oligonucleotide ERdj7
1298	ERdj7_guide2_S	CACCGACCACCCTAACATCCACTTT	CRISPR-Cas9 guide oligonucleotide ERdj7
1299	ERdj7_guide2_AS	AAACAAAGTGGATGTTAGGGTGGTC	CRISPR-Cas9 guide oligonucleotide ERdj7
1367	ERdj1_genomic_S	CCGGCGCGCTTACCTGC	Oligo for genotyping indels
1368	ERdj1_genomic_AS	CTCGGAACCCAGTGCGATGTG	Oligo for genotyping indels
1369	ERdj2_genomic_S	GCCATACAGTTGAATTTATTTATGTCATTTGTC	Oligo for genotyping indels
1370	ERdj2_genomic_AS	GTTAACAGTTCAAATTCTGATTTCTTTTGGAAATGTC	Oligo for genotyping indels
1371	ERdj3_genomic_S	AGTGGGGACTGTGAGAGAAGG	Oligo for genotyping indels
1372	ERdj3_genomic_AS	AATTTCTCCTGGGCTTGGGGATCG	Oligo for genotyping indels
1373	ERdj4_genomic_S	GTGCATAGCTTTTCGAATGCTGC	Oligo for genotyping indels
1374	ERdj4_genomic_AS	CTTCAGCATCAGGGCTCTTATTTTTG	Oligo for genotyping indels
1375	ERdj5_genomic_S	GTATCTTAATGTCACTTAAATAAGAACTTGC	Oligo for genotyping indels
1376	ERdj5_genomic_AS	CCAGATATTTAAAAGAGAAATTTTACCTACC	Oligo for genotyping indels
1377	ERdj6_genomic_S	CATTCAGGATTATGAAGCTGCTCAGG	Oligo for genotyping indels
1378	ERdj6_genomic_AS	ACGCTAAGGGCTCTCAGAATAACG	Oligo for genotyping indels
1379	ERdj7_genomic_S	TACGTGCCTTTAAGAGTATTGGGAAG	Oligo for genotyping indels
1380	ERdj7_genomic_AS	CACTGAAATGGCACATACACTGACC	Oligo for genotyping indels
1391	ERdj1_genomic_2_S	ATGTGGGCTCCCGGCTTCGGAC	Oligo for genotyping indels
1392	ERdj1_genomic_2_AS	TTACCTGCTGCACCCCGAGGAACTC	Oligo for genotyping indels
1393	ERdj2_genomic_S [5'-6FAM]	GCCATACAGTTGAATTTATTTATGTCATTTGTC	Oligo for genotyping indels (FAM)
1394	ERdj3_genomic_S [5'-6FAM]	AGTGGGGACTGTGAGAGAAGG	Oligo for genotyping indels (FAM)
1395	ERdj4_genomic_S [5'-6FAM]	GTGCATAGCTTTTCGAATGCTGC	Oligo for genotyping indels (FAM)
1396	ERdj5_genomic_S [5'-6FAM]	GTATCTTAATGTCACTTAAATAAGAACTTGC	Oligo for genotyping indels (FAM)
1397	ERdj6_genomic_S [5'-6FAM]	CATTCAGGATTATGAAGCTGCTCAGG	Oligo for genotyping indels (FAM)
1398	ERdj7_genomic_S [5'-6FAM]	TACGTGCCTTTAAGAGTATTGGGAAG	Oligo for genotyping indels (FAM)
1409	ERdj1_genomic_2_AS [5'-6FAM]	TTACCTGCTGCACCCCGAGGAACTC	Oligo for genotyping indels (FAM)
1618	ERdj8_guide1_S	CACCGAGGCATATAAGAAGCTCGCC	CRISPR guide targeting Ch ERdj8 (Dnajc16)
1619	ERdj8_guide1_AS	AAACGGCGAGCTTCTTATATGCCTC	CRISPR guide targeting Ch ERdj8 (Dnajc16)
1650	ERdj8_genomic_1_S	gagagATGgaggtgaaaaagctgagcgtctc	genotyping ERdj8 KOs in CHO cells
1651	ERdj8_genomic_1_AS	ATCATAATTCAGGCTTCAGGCACCTGCCTACTGC	genotyping ERdj8 KOs in CHO cells
1652	ERdj8_genomic_2_S	cagaaggaagagagATGgaggtgaaaaagctg	genotyping ERdj8 KOs in CHO cells
1653	ERdj8_genomic_2_AS	CAGGCTACTAGCTGAGCACCAATACTC	genotyping ERdj8 KOs in CHO cells
1675	ERdj8_genomic_2_AS[5'-6FAM]	[6FAM]CAGGCTACTAGCTGAGCACCAATACTC	Oligo for genotyping indels (FAM)

Table S3: Clones generated in this study, related to STAR methods

Gene	Description	Clone	Allele	Amino acid sequence (number shows amino acid position at which insert/deletion occured)
ERDJ1	Knock out	#15	1	…RIPQ13inRGM*
			2	…RIPQ13inSPAPGAAVVLRLAAAAVPAGGRGAGARLGERRPGVVRLGGRGAAQLLRVPRGAAGCFICRHQKSIS*
		#16	1	…RRPG18indelLXRCGLCEPHTNVGGRRGGHIIGCCCCSCWRPWGRCAPGRAETWSCSTWWKRCSSTSTSSSGCSRMLHLQTSEKHIVSFH*
			2	…LSSS23inRLAAAAVPAGGRGAGARLGERRPGVVRLGGRGAAQLLRVPRGAAGCFICRHQKSIS*
ERDJ2	Knock out	#9	1	...YNPY83inGSIKLGSWSNSSRN*
			2	...EYNP82delWILEQQ*
		#16	1	...YNPY83inGSIKLGSWSNSSRN*
			2	...QEYN81indelP*
ERDJ3	Knock out	#8	1	...VPRS35delQETSPTASS*
			2	...VPRS35delAKGHQKGLQETSPTASS*
		#11	1	…RSAS37inDFELQESYCAAMNDSINLVFYIPSILDLGTKTRXLNTYKGHQKGLQETSPTASS*
			2	...VPRS35delAGN*
ERDJ4	Knock out	#1	1	...ERQI42inTM*
			2	...ERQI42delRPFTN*
		#21	1	...LASK25inRATMIS*
ERDJ5	Knock out	#4	1	…GVSK44inNCK*
			2	...YSLL40del*
		#11	1	…RQAF55indelITIIKWTFL*
			2	…RQAF55inKEISTEVTS*
ERDJ6	Knock out	#3	1	...KAQR373inKNIMIEVSKKQKKYSMYSLISG*
			2	...EKAQ372inHLEFLAGHHRPAQHQEEPDRSPAVTETVTETRLL*
		#4	1	...QIRE366delGTETVTETRLL*
			2	…EKAQ372inRVTETVTETRLL*
ERDJ7	Knock out	#22	1	…LAPK23inVMYWAQCQAGHLPSLTSIGGVLGI*
			2	…SHYY13indelQGDFGQCMCHFSVSVFQLVE*
		#25	1	…LAPK23inGATALRPPSAPEWMLGW*
			2	…RLAP22delSGC*
ERDJ8	Knock out	#37	1	…YKKL52inSPGNGILTKIKTLELRTSSFRSARPMRYCPMRKRGQTMTTMAMPGRTRAIRSSSASTASAISMTTSISRSPFSTSPSIRSAGTRLMRSTCCTFHTT*
			2	…ASQA43delGNGILTKIKTLELRTSSFRSARPMRYCPMRKRGQTMTTMAMPGRTRAIRSSSASTASAISMTTSISRSPFSTSPSIRSAGTRLMRSTCCTFHTT*
		#41	1	...YKKL52insYGRAYHLAPVLQ*
			2	…IKKA48del*
IRE1	Knock out	NC6	1	…KLPFT1100delSQN*
			2	…KLPFT1100delSQN*
IRE1	Knock in	CV1	1	…KLPFTIPELVCASPSRSSDGILYM…
			2	…KLPFTIPELVCASPSRSSDGILYM…
		CV8	1	…KLPFTIPELVCASPSRSSDGILYM…
			2	…KLPFTIPELVCASPSRSSDGILYM…

Table S4: Repair template used to generate the endogenous Q105C, related to STAR methods

to introduce Q105C and C109S mutations.

GCTAGAAATAGTGTGGAGTGATCAGTATAAGTCATCTCATTTGAACTTCTTGCCCTATGGGTGTTGTC
CCCACTCTAGAGGTATCAAAACTGAGCCTGGACAAGTCAAAGAATTTACCAAGACCAAAGCTTCCAG
GAAGAGGTGTGATTTCCCAAGGATACTCCCTCAGCTGTGATAGTATCTGTCCTGGAGCACAGGAATC
CAGAGTGATTCCAGAAACATCTGGCAAAACTCTGGGCATTGTTCCCTCTTGCTGTCATTTTCCTTTTTC
TAAGGTTTAGTTTGAATGTAGGAGTGATAGGAAAATCCAAATAGTTAATACCTCTGGCTCTGTTTATT
AACACTACCAAGAAATCTTTTATCCCTCAGAGAAAAGGTAATAGTATAGTAGAAGCCTAGCAGCAAG
TAGAGCAGGTATCTGCACAGTTTCAGGGCCCGTTCCAAAAATAAAGTTCACCGAAACAAGCAGTTGT
CAAAAGGGCCTGTCAGAAGTCAGCTGTGGGCTCAGAAGAGGCAGTTGTATGATGAGATGTGTTGAA
ACGTTGGGACTTCTTGGAGTTTCTTTTCCGGGCACCTACCATAACTTCCTTTTTATAACCCAAAGACTC
TGTTTTCAGTAAAGAATTACTTACTGAAGAAAACATCAAAACTCAGACCTTTGTTTGCCCTTGTAACC
ATTCTCCTTCTCTTGAACCTTGACTTTCTTTTTTTTTTTTCCCAAGGCAGGGTTTCTCTGTAGCTTTGGA
GCCTGTCCTGGCACTCACTCTGGAGACCAGGCTGGCCTCGAACTCACAGAGATCCGCCTGCCTCTGC
CTCCCGAGTGCTGGGATTAAAGGCGTGCACCACCAACGCCCAGCTTGAACCTTGACTTACTTCTAAG
TTGCTTCTTTTCAGCATTAATGGTCCTCCCATGGTAAAAGGCATTGTCTACCTGGCTTGGATTTATCTT
TGGCTGGGAGTTGAAGACAGATTTCTTTCTCCCTTACAATACCTAAGACTGTGCAGCTTTTCACATTT
GTGTAATTTTCTCAGAAAAGGCTTGTGGCACCCTCAGGGAATACCGGGATGGGAGACCCGAGGGAC
CTGTGCATCCTATGTCAAATAAATGTGTTGGTGTACAGAGTTCAGAGGCTGGGGAGCCCAGAGGCT
GTTGGAAGTAGATGTGGAAACCTAGGATCTCCTCCGTAGGTTCCAACTAAAAGCCTCCATCTGCAGT
GTGCTTCTCTGTTTTTAGAACTGGGTAACAGAAGCAGCATGCTTAGTAAAATTACAGCAATTTCACAA
GACTTGGTTTACTTATTTTTTTACTTTTTTTTTTTAAAGAAACTTCCCTTTACCATCCCAGAATTAGTCT
GTGCTAGCCCATCCCGAAGTTCAGATGGAATCCTCTACATGGGTAAGAGTTCCCATTTTCAGCACCA
AAAACTTGGCTCTTTTGACAGTGAATCCTGGAATCTGGGAAAGTTGTGATTAAGAGAGCAGGATATT
GTCACCCTCTTTCAAATCTTTCTGAGAAAAGGTTTCCTTGCCTCAGTTGTGCTTTGGTGCTGCTGTTCA
TGCCAGGATCATCTGGCCAATTAAGAAAGAGTTCTCTCTGAGGAAAAATTGACATCATTAATATGGT
TACTCATTTAACAAGCTTTTAGCCATTTGCAACCTACCTAGCTGCAAATGGATAAAATTTAAAGTGAA
TACAGTAGTCTGAGCCAAGGAAGGGAAGCCAGGCCCTGCCTATTAATTACAGAGCTTTCTTAGGATT
TCCTGCTATCAGAATATTTGGTAGAAGCAGCACTTAACAGCTTGCCTAGATAAGCTGTCTTCATACCT
GTGGGGTCACAGCTTTCAAAGCATCAGCTCACCTGCTTTTCTTCAGCACATTGCTGCCAAAATGTCAA
TAAAAATGCATTGGTTGCACAAAGAAGAGCTGTGTCCTGTGTTTATATAGCAGCTTTCTTTTTGAGGA
ACTGAAAGTGCCTGTTGCCTATTCATGCCTCAGGAGCTCTATGTACATTTTCAAGTCTCAAATGCAGG
CCGGCTGGTTTTTAAGTTTTGCTAGGGACACCCAAGCTCCTGCTTCTTTGCACGAGCTTTTCAGAATG
AGGAACTCCCAACCAATATATTCGTAGGAAATGACCAGCAAACCTCTGGGCCATGCTCTGGACCCAT
ATGACCATCTTCTGTGTTATTGTTCTCTATCGGTCTTCCAGCAATCCATCATCTGTCATCAACTTAGGA
TATTTTGTTTCCACCATCTTTGTGTCATGTCTTAAGCTAGTTCACTGAGTATATTTATGAGCATGATAG
TTACTCAATCCACCCATCATAGCTGTAGGTAGATTATACAGACTGCAGTGGTCCTTGAAGTCACCAG
GTGTATGTCTCATAGATGGAAGGTATTAATGGTCATAGTTATTTGGATATATTTTATTCTCTATATTTA
GACCTTTTTAATTCTAAGACCTTATTGTTGAAAACAGTTAGGATCCACAAACAAGTTGGTCATGTGTG
GAATCTGGTGTATTACATCTGTTACAGCTAAGTATAGCTTTAGCAAATTCTGTTGCCCTGTGACCTTCT
CTGGCATCTCTGTATTCTACTTCCATAGGAACTTGAATGTAGGAAACATCTATTAAAGTAGGAAGATA
TAAATGTCTAATAAATGTCCAGAGGCGTGGCTTACAGAATAGAAATGCACACATTACAGTTGACTGT
GTTGGCCATCAAATATACCATACCATCCATACCGTAGACACATATGCTATATATTTTCCTAAAAGTGTT
TATGTGACTGTACTGTCTGTGTGTTTATATTTAATCAGCTGACCAAGGCAAGAGGTATAACCTGTGAC
CTGGGAATAGTT

<u>**Table S5: Protein concentrations used in this study, related to STAR methods**</u>

	Figure	Component	Conc in reaction	UK Reference Number
**Fig 4**				
	4C	IRE1LD-bio	5 µM	1915
		ERdj4	8 µM	2012
		BiP	30 µM	173
		ERdj4 QPD	8 µM	2040
		JERdj4	8 µM	2041
		BiP V461F	30 µM	182
		BiP T229A	30 µM	838
		ATP	2 mM	
	4D	IRE1LD-bio	5 µM	1915
		ERdj4	8 µM	2012
		BiP	30 µM	173
		ATP	2 mM	
	4F	IRE1LD-bio	n/a	1915
		IRE1LD-TAM	0.5 µM	2028
		ERdj4	8 µM	2012
		BiP	30 µM	173
		ERdj4 QPD	8 µM	2040
		JERdj4	8 µM	2041
		BiP V461F	30 µM	182
		BiP T229A	30 µM	838
		ATP	2 mM	
		ADP	2 mM	
	4G	IRE1LD-bio	n/a	1915
		IRE1LD-TAM	0.5 µM	2028
		ERdj4	8 µM	2012
		GRP170	1 µM	1264
		BiP	30 µM	173
		ATP	2 mM	
**Fig S3**				
	S3A	IRE1LD-bio	5 µM	1915
		ERdj4	4 µM	2012
		BiP	30 µM	173
		BiP NBD	30 µM	2008
		ATP	2 mM	
		ADP	2mM	
	S3B	IRE1LD-OG	0.7 µM	2047
		ERdj4	5 µM	2108
		BiP	40 µM	173
		ATP	2 mM	
**Figure 5**				
	5A	Ire1LD-bio	25 nM	1915
		GADD34-bio	25 nM	1920
		ERdj4	1.5 µM	2012
		ERdj4 QPD	1.5 µM	2040
		JERdj4	1.5 µM	2041
		BiP	1 µM	173
		BiP V461F	1 µM	182
		BiP T229A	1 µM	838
		ATP	2 mM	
	5B	Ire1LD-bio	200 nM	1915
		ERdj4	1.2 µM	2012
		BiP	6 µM	173
		BiP V461F	6 µM	182
	5D	IRE1LD-bio	n/a	1915
		IRE1LD-TAM	0.5 µM	2028
		ERdj4	8 µM	2012

	BiP	30 µM	173
	GRP170	1 µM	1264
	ATP	2 mM	

5E	IRE1LD-bio	25 nM	2007
	IRE1LD Q105C-bio	25 nM	2045
	ERdj4	2.5 µM	2012

5F	IRE1LD-biok	6 µM	2007
	IRE1LD Q105C-bioK	6 µM	2045
	ERdj4	8 µM	2012
	BiP	30 µM	173
	ATP	2 mM	

Figure S4

S4A	ERdj4-bio	200 nM	2098
	Ire1LD	varies	2007

S4B	Ire1LD-bio	25 nM	1915
	ERdj4	varies	2012
	BiP	1 µM	173
	ATP	2 mM	

S4C	Ire1LD-bio	25 nM	1915
	ERdj4	1.5 µM	2012
	BiP	varies	173
	BiP V461F	1 µM	182
	BiP T229A	1 µM	838
	ATP	2 mM	

S4D	Ire1LD-bio	2.3 uM	2007
	IRE1LD Q105C-bio	2.3 uM	2045
	ERdj4	1.7 µM	2012
	BiP	6 µM	173
	ATP	2 mM	

Figure 6

6A	Ire1LD-R234C	66 nM	2048
	Ire1LD-S112C	133 nM	2076
	Ire1LD	varies	2007
	BiP	varies	173

6B	Ire1LD-R234C	66 nM	2048
	Ire1LD-S112C	133 nM	2076
	BiP	50 µM	173
	ERdj4	30 µM	2108
	ERdj4 QPD	2.5 µM	2119
	JERdj4	2.5 µM	2041
	BiP AMP	30 µM	173
	BiP V461F	30 µM	182
	BiP T229A	30 µM	838
	GRP170	1 µM	1264

6C	Ire1LD-R234C	66 nM	2048
	Ire1LD-S112C	133 nM	2076
	BiP	50 µM	173
	ERdj4	2.5 µM	2012
	JERd6	2.5 µM	185

Figure S5

S5	Ire1CLD-R234C	66 nM	2117
	Ire1CLD-S112C	133 nM	2118
	BiP	50 µM	173
	ERdj4	2.5 µM	2012
	ATP	2 mM	

Table S6: Reproducibility of key observation, related to STAR methods

Figure		Number of times key observation has been reproduced	
1	A	3	with 2 independent clones
	B	3	
	C	>3	
	D	3	
	E	6	
	F	n/a	
S1	A	3	
	B	n/a	
	C	3	
	D	>3	
2	A	n/a	
	B	>3	
	C	n/a	
	D	3	
	E	3	
	F	6	
	G	3	
	H	>3	
3	A	n/a	
	B	2	
	C	3	
	D	n/a	
	E	6	
	F	n/a	
S2	A	>3	with 2 independent clones and mixed population
4	A	n/a	
	B	n/a	
	C	>3	twice in its entirety
	D	2	
	E	n/a	
	F	>3	twice in its entirety
	G	3	
	H	n/a	
S3	A	2	
	B	>3	
	C	n/a	
5	A	>3	
	B	2	
	C	n/a	
	D	4	
	E	>3	
	F	2	
S4	A	>3	
	B	>3	
	C	>3	
	D left	>3	
	D right	2	
6	A	>3	
	B	>3	
	C	>3	
S5	A	>3	
7	A	n/a	
